# Endosomal RFFL ubiquitin ligase regulates mitochondrial morphology by targeting mitofusin 2

**DOI:** 10.1242/jcs.263830

**Published:** 2025-06-20

**Authors:** Nikhil Dev Narendradev, Rishith Ravindran, Parul Jain, Shikha Chaudhary, Anoop Kumar G. Velikkakath, Abyasree Sudharman, Adithya Janardhanan, Tapas Chandra Nag, Subhash Chandra Yadav, Srinivasa Murty Srinivasula

**Affiliations:** ^1^ School of Biology, Indian Institute of Science Education and Research Thiruvananthapuram, Thiruvananthapuram 695551 Kerala, India; ^2^ Electron Microscope Facility, Department of Anatomy, All India Institute of Medical Sciences, 110029 New Delhi, India; ^3^ Center for Systems Biology and Molecular Medicine, Yenepoya Research Centre, Yenepoya University, Deralakatte 575018, Mangalore, India

**Keywords:** E3 ubiquitin ligase, RFFL, Endosomes, Mitochondrial morphology, Mitofusin 2, Ubiquitylation

## Abstract

Mitochondrial homeostasis is ensured through communication between diverse cellular organelles, including mitochondria, the endoplasmic reticulum (ER), lysosomes and endosomes. Although it is known that mitofusins regulate mitochondrial networks and ER contacts, their role in endosomal–mitochondrial interactions remains unclear. Previously, we have reported that vesicles positive for the endosomal ubiquitin ligase RFFL are associated with damaged mitochondria and prime the organelle for PRKN recruitment. Now, we establish that RFFL is a ubiquitin ligase for mitofusin 2 (MFN2). Using electron microscopy and confocal imaging analyses, we demonstrate that RFFL-knockout cells exhibit enlarged mitochondrial morphology. RFFL interacts at an endogenous level with MFN2 and contributes to its ubiquitylation upon mitochondrial damage. Recombinant RFFL interacts and ubiquitylates MFN2 protein *in vitro*. Furthermore, exogenous RFFL, in a ligase-dependent manner, specifically reduces the exogenous protein levels of both MFN1 and MFN2, but not that of DRP1, and also perturbs lipid homeostasis. Importantly, we show that the hyperfused mitochondria morphology reported with expression of pathogenic disease mutants of MFN2 (T206I and R364W) of Charcot–Marie–Tooth disease type 2A can be rescued by RFFL co-expression. The study unravels novel mechanisms involving endosomal ubiquitin ligases in mitochondrial networks.

## Teaser

**Highlighted Article:** The endosomal ubiquitin ligase RFFL associates with and primes mitochondria for PRKN recruitment, targets mitofusins for degradation and contributes to mitochondrial networks and lipid homeostasis.

## INTRODUCTION

Mitochondria are motile and dynamic organelles that play crucial roles in diverse cellular processes, including ATP generation, metabolism and Ca^2+^ signaling (Cosentino and García-Sáez, 2014; McBride et al., 2006; Nunnari and Suomalainen, 2012). The dynamics of these organelles encompass changes in their morphology, degradation, fission and fusion (Dorn, 2019; Quintana-Cabrera and Scorrano, 2023). Although distinct signaling events contribute to the dynamics, dysregulation of the same has been reported in numerous pathological conditions in humans (Knott et al., 2008; Youle and van der Bliek, 2012). Whereas diseases that are associated with defects in mitochondrial dynamics range from neurodegenerative disease to cancer, primary mitochondrial dysfunction typically affects the brain, heart and skeletal muscles (Knott et al., 2008; Ng and Turnbull, 2016).

One of the major proteins involved in mitochondrial dynamics is mitofusin 2 (MFN2), an outer membrane mitochondrial GTPase. Mitofusins present on opposing mitochondria via trans interactions (homo or hetero oligomeric complex formation) are believed to mediate tethering between the organelle during fusion (Chen and Chan, 2005). The 757-amino-acid long MFN2 consists of a GTPase domain, two coiled-coil domains (HR1 and HR2) and a proline-rich domain (PR) between the HR1 and transmembrane domains (TM) (Filadi et al., 2018). The GTPase and HR1 domains are in the cytosol, whereas the TM spans the outer mitochondrial membrane, and HR2 and C-terminal reside within the intermembrane space (Mattie et al., 2017). Although MFN2 knockout in mice is embryonic lethal, mutations in MFN2 are associated with multiple disorders, including Charcot–Marie–Tooth disease type 2A (CMT2A), a peripheral neuropathy disease (Cartoni et al., 2010; Chen et al., 2003; Sandoval et al., 2014), along with atherosclerosis and hypertension (Chen et al., 2004; Guo et al., 2007; Shen et al., 2007), exemplifying the importance of MFN2 for cellular homeostasis.

Diverse molecules involved in mitochondrial dynamics are regulated by post-translational modifications, such as phosphorylation, acetylation and ubiquitylation (Chen et al., 2023), including MFN2. Phosphorylation of MFN2 at T111 and S442 by PINK1 facilitates PRKN binding and subsequent ubiquitylation of MFN2 (Chen and Dorn, 2013). MFN2 is also reported to be ubiquitylated by other E3 ligases (Escobar-Henriques and Joaquim, 2019) apart from PRKN (Gegg et al., 2010; Poole et al., 2010; Tanaka et al., 2010; Ziviani et al., 2010), like membrane associated RING-CH-type finger 5 (MARCHF5; also known as MARCH5 and MITOL) and mitochondrial E3 ubiquitin protein ligase 1 (MUL1) (Kim et al., 2015; Li et al., 2008; Sugiura et al., 2013; Tang et al., 2015; Yun et al., 2014). Although PRKN is a cytosolic ubiquitin ligase, MARCHF5 and MUL1 are mitochondrial E3 enzymes. Apart from these, two more cytosolic E3 ligases, RANBP2-type and C3HC4-type zinc finger containing 1 (RBCK1; also known as HOIL1) and HECT, UBA and WWE domain containing e3 ubiquitin protein ligase 1 (HUWE1) have been shown to ubiquitylate MFN2 (Di Rita et al., 2018; Leboucher et al., 2012; Michel et al., 2017; Su et al., 2024). Furthermore, AMFR (also known as GP78), an E3 ligase associated with the ER-associated degradation pathway, also increases MFN2 ubiquitylation and degradation, although direct interaction between these molecules has yet to be demonstrated (Fu et al., 2013; Shankar et al., 2013). Given the links of mitofusins and post-translational modifications with diverse cellular processes, including mitophagy, hypoxic and genotoxic stress, Ca^2+^ signaling, and mitochondrial transport, it is understandable that regulatory pathways of mitofusins by more E3s localized to different compartments are waiting to be discovered.

RFFL (also known as CARP2, RNF189, RNF34L, RIFIFYLIN and Sakura) was initially identified as a palmitoylated E3 ligase containing a RING finger domain in the brain, testes, and endocrine organs alongside its homolog RNF34 (also known as CARP1) (Araki et al., 2003). This discovery was concurrently complemented by the work of McDonald and El-Deiry, who identified RFFL and RNF34 as interacting partners of the death effector domain (DED) of caspase-8 and -10 in yeast two-hybrid screen (McDonald and El-Deiry, 2004). Both RFFL and RNF34 also contain an FYVE-like domain (a cysteine-rich zinc finger found in Fab1, YOTB, Vac1, and EEA1 proteins), which confers the ability to bind phosphatidylinositol 3-phosphate in conjunction with modifications like palmitoylation (Ravindran et al., 2022; Tibbetts et al., 2004). RFFL has been shown to localize to intracellular vesicles, such as Rab5B- and Rab7A-containing endosomes, the plasma membrane and the Golgi (Coumailleau et al., 2004; Liao et al., 2008; Okiyoneda et al., 2018; Ravindran et al., 2022; Sakai et al., 2019). Diverse signaling molecules like the receptor-interacting serine/threonine kinase 1 (RIPK1), tumor protein p53 (TP53), caspase 8 and 10, proline rich 5 like (PRR5L), STUB1, and K^+^ voltage-gated channel subfamily H member 2 (KCNH2; also known as hERG) are targeted by RFFL for ubiquitylation (Gan et al., 2012; Liao et al., 2008; McDonald and El-Deiry, 2004; Roder et al., 2019; Sharma et al., 2023; Yang et al., 2007). Additionally, RFFL is involved in protein quality control, specifically targeting misfolded mutant cystic fibrosis transmembrane conductance regulator (CFTR) protein for ubiquitylation and proteasomal degradation, as shown in research by Okiyoneda et al. (2018).

Earlier studies from our group have demonstrated that RFFL-positive endosomes are associated with damaged mitochondria prior to PRKN recruitment and facilitate the clearance of mitochondrial proteins. RFFL-knockout cells exhibited a delay in the translocation of PRKN to fragmented mitochondria, suggesting an important role for RFFL in maintaining mitochondrial homeostasis (Ravindran et al., 2022). However, no information on the morphology of mitochondria in RFFL KO cells and whether RFFL targets any mitochondrial proteins is available. In this paper, we include evidence from electron micrograph images that show the enlargement of mitochondria in RFFL KO cells. We further demonstrate that RFFL interacts with and ubiquitylates MFN2, resulting in its degradation in cells. Our study, for the first time, shows direct ubiquitylation of MFN2 by RFFL *in vitro*, using recombinant proteins. Importantly, our findings also unravel hitherto unknown cross-talk between endosomal ubiquitin ligases and MFN2 and further MFN2 biology.

## RESULTS

### RFFL affects mitochondrial morphology

**Fig. 1.**
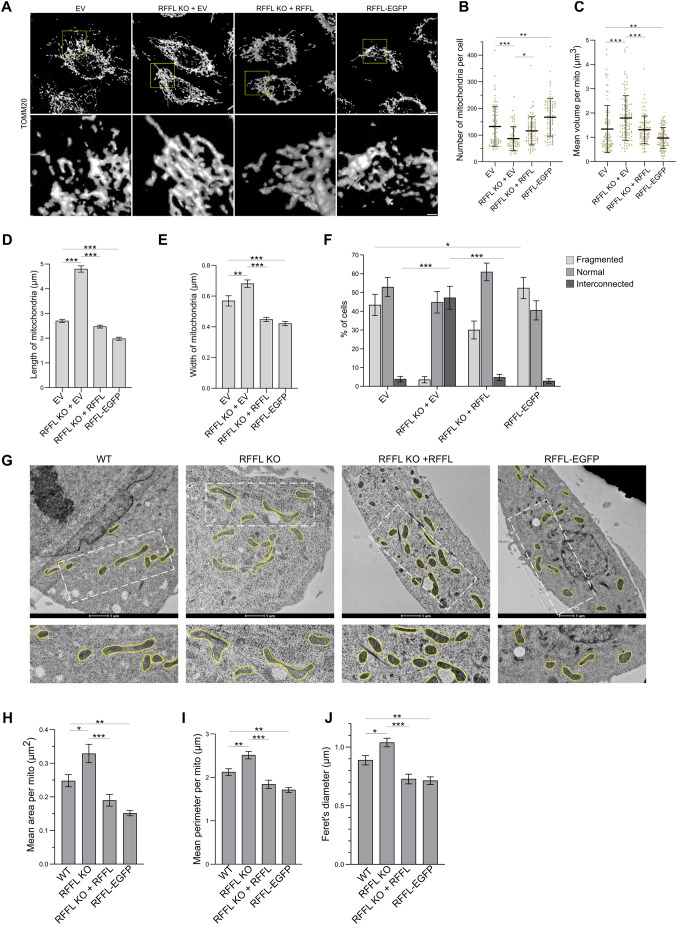
**RFFL controls mitochondrial morphology.** (A) A549 cells expressing empty vector (EV) or RFFL–EGFP, or A549 RFFL KO cells stably expressing EV (RFFL KO+EV) or untagged WT RFFL (RFFL KO+RFFL) were immunostained with anti-TOMM20 antibody, and multiple *Z*-planes were acquired with confocal microscopy. Deconvolution of images was performed, and representative 3D surface rendering is shown. Full 3D volume visualization is provided in Movies 1–4. The contrast was adjusted uniformly across the images for better visibility. Scale bars: 5 µm (main images); 1 µm (inset). (B,C) Quantification of results as in A showing the number of mitochondria per cell and mean volume of a mitochondrion in a cell measured using the mitochondria analyzer plugin. Error bars represent mean±s.d. The adjusted *P*-value for the RFFL KO+EV versus RFFL KO+RFFL is 0.0289, and EV versus RFFL–EGFP is 0.0023 in B and the adjusted *P*-value for EV versus RFFL–EGFP is 0.0075 in C. (D,E) Quantification of results as in A showing length and width of mitochondria measured manually (mean±s.e.m.). The adjusted *P*-value for EV versus RFFL KO+EV in E is 0.0033. (F) Graph showing the percentage of cells with mitochondria in the indicated categories (mean±s.e.m). Adjusted *P*-values for EV versus RFFL–EGFP is 0.042 (fragmented) and for others is *P*<0.001. In B–F, all quantification was done from three independent experiments with a minimum of 70 cells. (G) Representative TEM images showing mitochondria from A549 WT, A549 RFFL KO cells (RFFL KO), A549 RFFL KO cells stably reconstituted with RFFL without any tag (RFFL KO+RFFL) and A549 cells stably expressing RFFL–EGFP (RFFL–EGFP). Yellow lines mark the boundary of mitochondria (manually annotated). Scale bars: 1 µm. (H–J) The area, perimeter and Feret's diameter of a mitochondrion quantified from TEM images. A minimum of 45 mitochondria were marked and quantified from different cells in three independent experiments for TEM imaging. Error bars represent mean±s.e.m. In the case of H, the adjusted *P*-value for WT versus RFFL–EGFP is 0.0021, and for WT versus RFFL KO is 0.0110. In the case of I, the adjusted *P*-value for WT versus RFFL–EGFP is 0.0076, and WT versus RFFL KO is 0.0084. In the case of J, adjusted *P*-value for WT versus RFFL–EGFP is 0.0079, and WT versus RFFL KO is 0.0197. For all panels, *P<0.05; **P<0.01; ***P<0.001 (unpaired two-tailed Student's *t*-test with Bonferroni's correction).

The delayed PRKN recruitment to mitochondria and reduced elimination of mitochondrial proteins observed in RFFL-knockout cells (Ravindran et al., 2022) led us to closely evaluate mitochondrial networks in these cells using confocal microscopy after immunostaining with anti-TOMM20 (one of the outer mitochondrial proteins) antibody and transmission electron microscopy (TEM) because a similar phenotype has been reported in cells with hyperfused mitochondria (Gomes et al., 2011; Rambold et al., 2011). Deconvoluted three-dimensional (3D) images revealed more enlarged or fused mitochondrial networks in A549 RFFL-knockout (KO) cells than in wild-type (WT) cells. Importantly, this phenotype was reversed when RFFL KO cells stably expressed RFFL (reconstituted cells) (Fig. 1A; Movies 1–4). Expression levels of RFFL protein in these cells are shown in Fig. S1A. To evaluate these differences, we measured different parameters, such as the number of mitochondria per cell, the mean volume of mitochondria, and the length and width of mitochondria using methods described previously (Chaudhry et al., 2020; Das et al., 2022a,b; Triolo et al., 2023). Data from these measurements (Fig. 1B–E) confirmed significant differences in mitochondria in cells with or without RFFL expression. Furthermore, we also measured the average length of mitochondria in each cell and categorized them as fragmented, normal or interconnected/hyperfused mitochondria as described previously (Das et al., 2022a; Parra et al., 2008) and plotted the results (Fig. 1F). Again, the data showed a significant increase in interconnected mitochondria in cells that did not express RFFL compared to in the RFFL-expressing cells (Fig. 1F).

We further examined mitochondria in these cells using transmission electron microscopy (TEM) (Fig. 1G). Consistent with the observations from the confocal microscopy images, in RFFL KO cells, mitochondria appeared to be enlarged compared to those in control WT cells. This phenotype was the result of RFFL loss, as mitochondria from KO cells stably reconstituted with RFFL showed morphology similar to that observed in WT cells (Fig. 1G). Conversely, the mitochondria in A549 cells stably expressing RFFL–EGFP appeared to be shorter in length. When the mitochondrial area, perimeter and Feret's diameter from these images were measured, they were found to be significantly higher in RFFL KO cells compared to in WT cells and the KO cells reconstituted with RFFL. Consistent with this, the same parameters of mitochondria in cells stably expressing RFFL–GFP were also significantly lower than that in WT cells alone, suggesting RFFL expression influences the extent of defect in mitochondrial morphology assessed by various parameters measured (Fig. 1H–J; Fig. S1A). These various mitochondrial parameters quantified fall within the ranges reported previously, indicating the robustness of the image analysis (Faitg et al., 2020; Han et al., 2021; Parra et al., 2013; Sidarala et al., 2022). The marking of the mitochondria used for measurements is shown in Fig. S1B. Together, the data shows that mitochondrial morphology is influenced by RFFL.

### RFFL vesicles associate with MFN2 and affect MFN2 modification

**Fig. 2.**
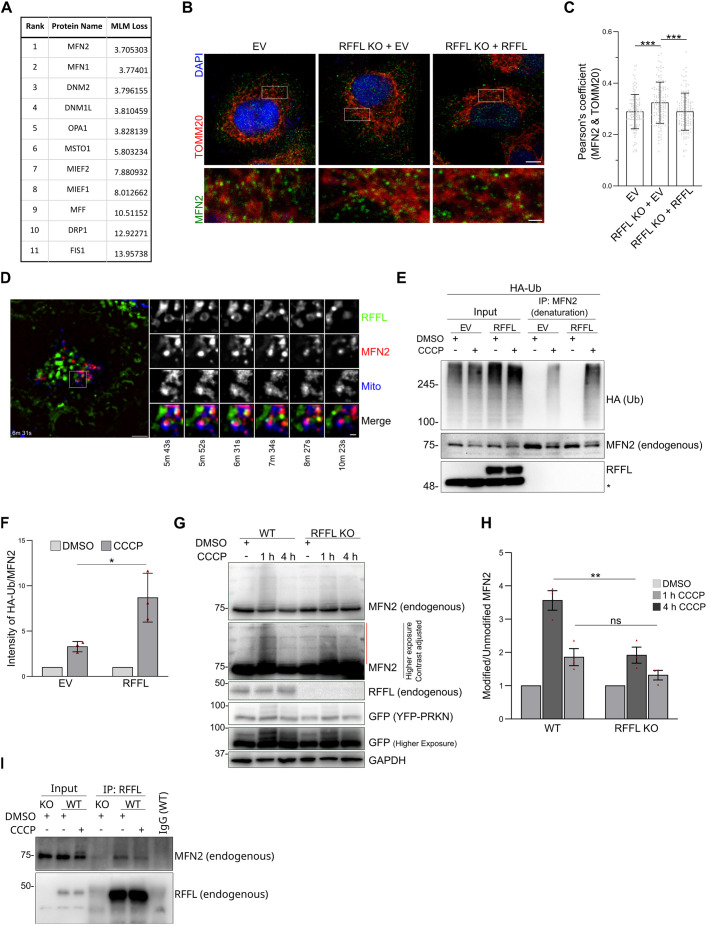
**MFN2 modification is affected by RFFL.** (A) Proteins known to be involved in mitochondrial morphology were considered as potential substrates of RFFL. Among these proteins, interaction probability with RFFL was calculated using the protein language model, and a ranked list with MLM loss values is shown. (B) A549 cells expressing EV, A549 RFFL KO cells stably expressing with EV (RFFL KO+EV) or untagged WT RFFL (RFFL KO+RFFL) were immunostained with anti-TOMM20 (Red) and anti-MFN2 antibody (green) and imaging was done with confocal microscopy. Scale bars: 5 µm (main images); 1 µm (inset). (C) Quantification of B showing Pearson's coefficient for MFN2 and TOMM20. Error bars represent mean±s.d. from four independent experiments with a minimum of 20 cells per experiment. Statistical significance was calculated using unpaired two-tailed Student's *t*-test with Bonferroni's correction. (D) A549 cells stably expressing RFFL–EGFP were transfected with MFN2–iRFP670, and mitochondria were stained with MitoTracker Red CMXRos. Live time-lapse imaging was performed, and snapshots are shown here. Images shown representative of three experimental repeats. Scale bars: 5 µm (main image); 1 µm (inset). (E) HEK293T cells were transfected with HA–Ub for 24 h and then treated with DMSO or 10 μM CCCP for 45 min, and endogenous MFN2 was immunoprecipitated (IP) under denaturing conditions. * represents a non-specific band recognized by anti-RFFL antibodies. Input, 3%. (F) The bar graph shows the ratio of the intensity of the HA signal to the MFN2 signal in IP samples of from experiments as in E plotted as mean±s.d. from three independent experiments. CCCP values are normalized to DMSO values. *P*=0.0273 (unpaired two-tailed Student's *t*-test). (G) Extracts from A549 (WT or RFFL KO) stably expressing YFP–PRKN cells that were treated with DMSO or 10 μM CCCP at different time points were immunoblotted for the indicated proteins. Images from both shorter and longer exposure times were included. The red line indicates modified MFN2. (H) Bar graph showing the ratio of the modified and unmodified bands of endogenous MFN2 from experiments as in G. Values were normalized with DMSO samples. Error bars represent mean±s.d. from three independent experiments. *P*=0.007 and ns=0.887 (unpaired two-tailed Student's *t*-test with Bonferroni's correction). (I) A549 WT or RFFL KO cells stably expressing YFP–PRKN were treated with either DMSO or CCCP (20 μM) for 2 h, and endogenous RFFL was immunoprecipitated using antibody against RFFL. For IgG control, mixed lysate from A549 untreated and treated was used. Blot shown representative of three experimental repeats. For all panels, *P<0.05; **P<0.01; ***P<0.001; ns, not significant.

Given that mitochondria appear different in RFFL KO cells, we hypothesized that proteins involved in mitochondrial dynamics could be potential substrates for RFFL. A number of proteins have been reported to mediate mitochondrial fusion and fission (Chen et al., 2023). To predict potential substrates for RFFL among these proteins, we applied a previously reported protein language model. This model ranked the proteins based on their likelihood of being RFFL substrates by evaluating the masked language modeling (MLM) loss for each protein pair. A lower MLM loss when analyzing protein pairs indicates that the model identifies a coherent or complementary context between them, suggesting a possible interaction (Lin et al., 2023; Lupo et al., 2024). Among the tested proteins, the model ranked MFN2 and MFN1 as the most likely interacting partners of RFFL (Fig. 2A). Interestingly, the hyper-fused mitochondria observed in RFFL KO cells appeared to be very similar to the previously reported phenotype of mitochondria observed in midbrain neurons and brain tissues of Mfn2 transgenic mice (Han et al., 2021; Zhao et al., 2017). This led us to first test whether MFN2 could be a potential substrate for RFFL. The role of MFN2 is well-established in mitochondrial biology, and the localization of MFN2 to mitochondria facilitates fusion (Dorn, 2020; Koshiba et al., 2004). Hence, we evaluated MFN2 localization to mitochondria with and without RFFL in cells by immunostaining with anti-MFN2 antibody and anti-TOMM20 (for mitochondria). Confocal imaging and Pearson's correlation coefficient analyses revealed increased MFN2 colocalization with TOMM20 in RFFL KO cells (Fig. 2B,C). Furthermore, we evaluated the dynamics of RFFL, MFN2 and mitochondria in live cells using confocal imaging. For this, we expressed MFN2–iRFP670 in A549 cells stably expressing RFFL–EGFP and stained the mitochondria with MitoTracker Red CMXRos. As expected, the clustering of mitochondria as a result of MFN2 overexpression was observed (Fig. 2D; Fig. S2A) (Huang et al., 2007). Monitoring of live cells showed an association of RFFL-positive vesicles with MFN2 and the mitochondrial population (Fig. 2D; Fig. S2A, Movies 5, 6). Interestingly, RFFL vesicle–mitochondria contact sites were also found to be positive for MFN2. This association, although transient, appears consistently in various mitochondria (Movies 5 and 6). These observations demonstrate the presence of RFFL vesicles in the close vicinity of MFN2-labeled mitochondria, even under constitutive conditions. Given that RFFL vesicles appear to come into contact with MFN2 on mitochondria, we hypothesized that RFFL can target MFN2 for ubiquitylation. As MFN2 is known to undergo rapid ubiquitylation during PRKN-mediated mitophagy triggered by mitochondrial depolarization (McLelland et al., 2018), we first investigated the effect of RFFL on this ubiquitylation process.

For this, we transfected HEK293T cells stably expressing empty vector (EV) or RFFL with HA–ubiquitin (Ub) and treated the cells with mitochondrial depolarizing reagent carbonyl cyanide 3-chlorophenylhydrazone (CCCP) or DMSO. The endogenous MFN2 was immunoprecipitated under denaturing conditions using an anti-MFN2 antibody, and the precipitates were immunoblotted with an anti-HA antibody. As expected, upon mitochondrial damage conditions, a ubiquitin smear on MFN2 was observed. Importantly, an increased ubiquitin signal was noted in extracts from cells expressing RFFL exogenously compared to controls (Fig. 2E). The extent of ubiquitin modification of MFN2 was calculated and plotted (Fig. 2F). These results indicate that RFFL contributes to MFN2 ubiquitylation in cells. We also checked MFN2 ubiquitylation in the presence of PRKN and found that RFFL was able to increase the ubiquitylation of MFN2 in the presence of PRKN as well (Fig. S2B,C). Next, we investigated whether endogenous RFFL could also contribute to the modification of endogenous MFN2, which has been reported to occur upon treatment with CCCP (McLelland et al., 2018). For this, we assessed the extent of MFN2 modification in A549 WT and RFFL KO cells. As expected, treatment of cells with CCCP resulted in substantial modification of MFN2, appearing as a high molecular mass smear within 1 h of treatment. Importantly, much less MFN2 smear was observed in RFFL KO cells than in WT cells, demonstrating that RFFL contributes significantly to MFN2 modification (Fig. 2G,H). To further explore whether MFN2 forms a complex with RFFL in cells, we immunoprecipitated endogenous RFFL from extracts of WT or KO cells and immunoblotted for endogenous MFN2 (Fig. 2I). Consistent with the changed mitochondrial morphology observed in WT versus KO cells without any treatment (Fig. 1), an RFFL–MFN2 complex was noted in cells treated with DMSO, indicating an interaction between RFFL and MFN2 even under constitutive conditions. A slight reduction in MFN2 associated with RFFL after CCCP treatment was noted, suggesting perhaps enhanced modification and rapid turnover of MFN2 under stress conditions (Fig. 2I) (McLelland et al., 2018). These results collectively demonstrate that RFFL forms a complex with MFN2 and contributes to MFN2 ubiquitylation.

### RFFL directly Interact with MFN2

**Fig. 3.**
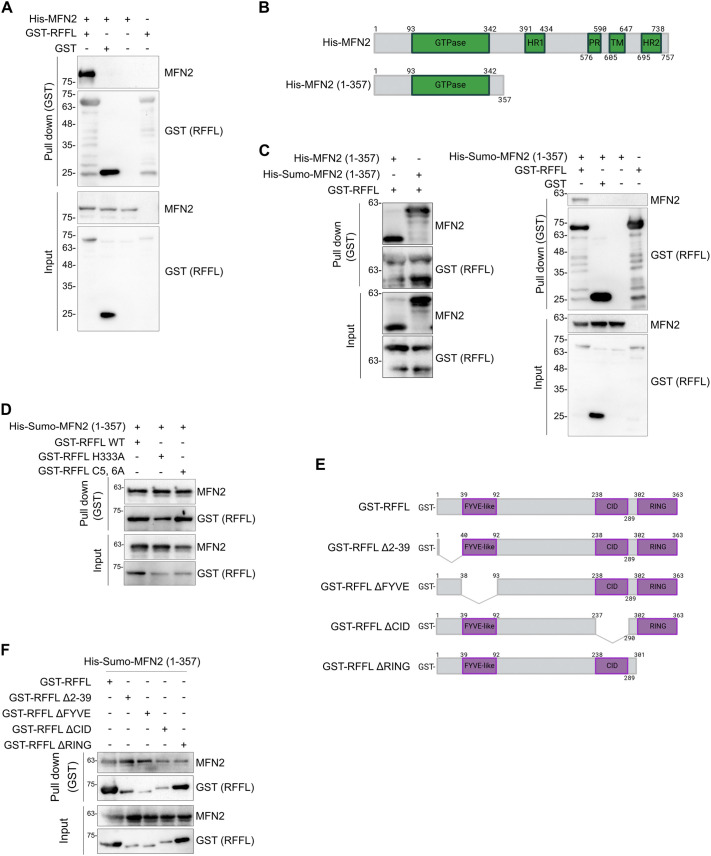
**RFFL directly interacts with MFN2**. (A) GST pulldown of extracts from bacterial cells expressing the indicated proteins were immunoblotted with indicated antibodies (see Materials and Methods). (B) Schematic representation of MFN2 variants. (C,D) GST pulldown of extracts from bacterial cells expressing indicated proteins were immunoblotted with indicated antibodies. Input, 1%. (E) Schematic representation of different RFFL deletion constructs used in the study. (F) GST pulldown of extracts from bacterial cells expressing indicated proteins were immunoblotted with indicated antibodies. Input, 1%. Blots shown representative of three experimental repeats.

Given that endogenous RFFL interacts with endogenous MFN2 (Fig. 2I) and the presence of MFN2 on RFFL-positive vesicles (Fig. 2D), we investigated the plausibility of a direct interaction between recombinant RFFL and MFN2. We co-expressed full-length RFFL with a GST tag and MFN2 with either a His or His–Sumo tag in *Escherichia coli* cells and used the extracts from these cells for pulldown using glutathione–Sepharose beads. Pulldown of GST–RFFL revealed the presence of His–MFN2, whereas no MFN2 was observed with pulldown of either GST alone or with beads (Fig. 3A). These results confirm that RFFL interacts directly with full-length MFN2. To identify regions in MFN2 interacting with RFFL, we co-expressed GST-tagged RFFL with truncated MFN2 (amino acids 1–357) with either His or His–Sumo tags (Fig. 3B). The binding of RFFL was still observed when the MFN2 (1–357) variant was used, suggesting that RFFL recognizes the cytosolic N-terminal of MFN2, which contains the GTPase domain. Whether His or His–Sumo was used as the tag made little difference between the interaction of tagged MFN2 (1–357) and GST–RFFL (Fig. 3C), with GST alone showing no interaction. Given that GST–RFFL interacts with the MFN2 (1–357) variant and there was not much difference in this interaction with His alone or His–Sumo tag, we used His–Sumo tagged MFN2 (1–357) for most of the other experiments, considering its better solubility. Next, we assessed the interaction of MFN2 with different variants of RFFL. The ubiquitin ligase activity of the RING domain-containing E3s is mediated by the coordination of a zinc ion by conserved cysteine and histidine residues. Substitution of any of these amino acids, for example, H333A in the case of RFFL, results in loss of the E3 activity (Araki et al., 2003; Freemont, 2000; Liao et al., 2008; Okiyoneda et al., 2018). Another variant, where the amino acids cysteine at 5 and 6 of RFFL (presumably involved in palmitoylation) are replaced by alanine, changes RFFL localization from intracellular vesicles to cytosolic (Araki et al., 2003; Okiyoneda et al., 2018; Ravindran et al., 2022). We found that both of these variants of RFFL interacted well with MFN2 (1–357) (Fig. 3D), suggesting that these mutations have no effect on MFN2 interaction *in vitro*. Next, we examined various constructs of RFFL with individual domains deleted. The deletion variants tested showed variations in the extent of binding, suggesting that MFN2 associates with RFFL through more than one domain (Fig. 3E,F), apparently with different strengths.

### Direct ubiquitylation of MFN2 by RFFL

**Fig. 4.**
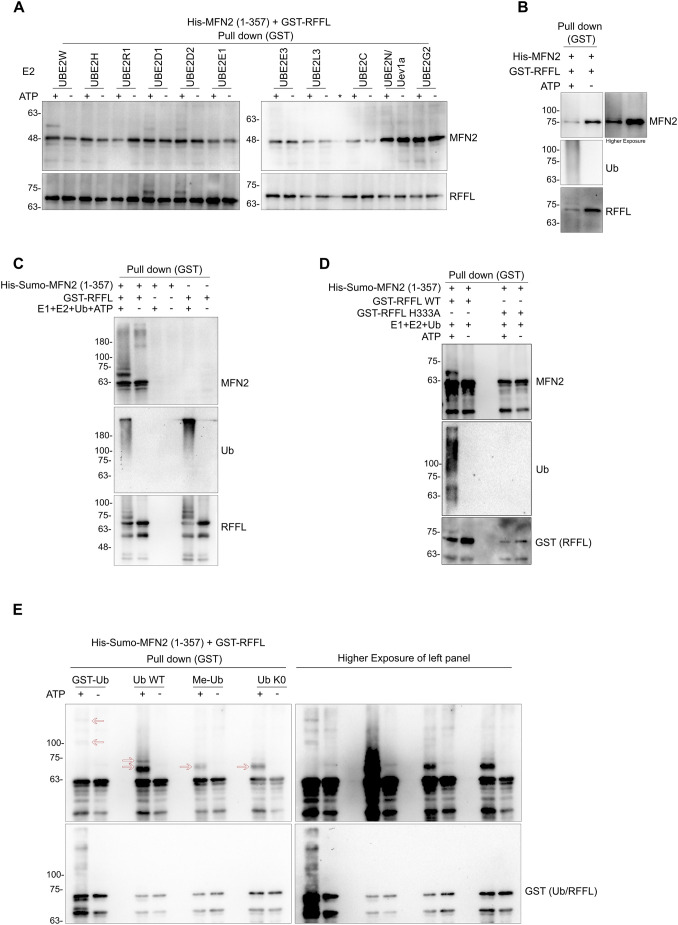
**RFFL ubiquitylates MFN2 *in vitro***. (A) His–MFN2 (1–357) and GST–RFFL were co-expressed in *E. coli* ArcticExpress cells, and the complex was pulled down using glutathione–Sepharose beads. An *in vitro* ubiquitylation assay was performed on these beads using indicated E2 enzymes. The reaction samples were heated in the SDS loading buffer and immunoblotted with indicated antibodies. Spillover lane was marked with *. (B) Same as A, but with full length His–MFN2 and UBE2D1 as E2. (C) Same as A, but with His–Sumo–MFN2 (1–357) and UBE2D1 as E2. (D) *In vitro* ubiquitylation assay with RFFL WT or H333A. (E) *In vitro* ubiquitylation assay using RFFL WT and His–Sumo MFN2 (1–357) and indicated ubiquitin variants. Blots shown representative of three experimental repeats.

Given that recombinant RFFL protein was found to directly interact with MFN2, we tested whether this interaction could lead to ubiquitylation of MFN2 *in vitro*. For this, we conducted a ubiquitylation assay using recombinant RFFL and MFN2. We co-purified MFN2 and RFFL and set up an *in vitro* ubiquitylation reaction on beads. As E2 enzymes also play a crucial role in substrate ubiquitylation along with E3s, we first evaluated the MFN2 ubiquitylation in the presence of different E2 enzymes and RFFL as E3 ligase. The results showed that only in the presence of the E2s UBE2W, UBE2D1 and UBE2D2 (also known as UBC16, UBCH5A and UBCH5B, respectively) ubiquitin modification on MFN2 by RFFL was observed (Fig. 4A). Whereas UBE2D1 and UBE2D2 promoted autoubiquitylation of RFFL as expected (Liao et al., 2008), the reaction with UBE2W did not result in RFFL modification. As expected, all these three E2s promoted MFN2 ubiquitylation in the presence of ATP only. None of the other eight E2s showed any appreciable modification of either RFFL or MFN2 (Fig. 4A). To our knowledge, this is the first report of MFN2 ubiquitylation by any of the E3s *in vitro*. We also found that full-length MFN2 is also ubiquitylated *in vitro* by RFFL, albeit to a lesser degree compared to the MFN2 (1–357) variant (Fig. 4B). To further validate the ubiquitylation of MFN2, we repeated the experiments with His–Sumo constructs, the results of which clearly demonstrated modification and a high molecular mass smear representing polyubiquitylation (Fig. 4C; Fig. S3A). Moreover, no such modification or smear was noted when E3 inactive RFFL was used (Fig. 4D), suggesting that the modification observed in the *in vitro* reaction was indeed a result of the ubiquitin ligase activity of RFFL.

Given that the ubiquitylation data from the *in vitro* assay showed the presence of multiple high molecular mass bands with MFN2-specific antibodies, we evaluated whether these modifications involve polyubiquitylation. To assess this, we used the ubiquitin variants Me-Ub (free amino groups of lysine residues are chemically modified by reductive methylation and hence cannot participate in further ubiquitin chain formation) (Kirisako et al., 2006) or Ub-K0 (all lysine mutated to arginine residues) along with GST–Ub (N-terminus of Ub is fused with GST tag) and untagged Ub (Ub–WT). Consistent with the idea that RFFL promotes polyubiquitylation, Ub-K0, and Me-Ub showed very few multiple bands of MFN2 compared to untagged WT Ub or GST–Ub in the presence of ATP (Fig. 4E, marked by arrows). The data collectively suggest RFFL targets MFN2 for polyubiquitylation, which might lead to its proteasomal or lysosomal degradation in cells.

### RFFL-mediated ubiquitylation targets MFN2 for degradation

**Fig. 5.**
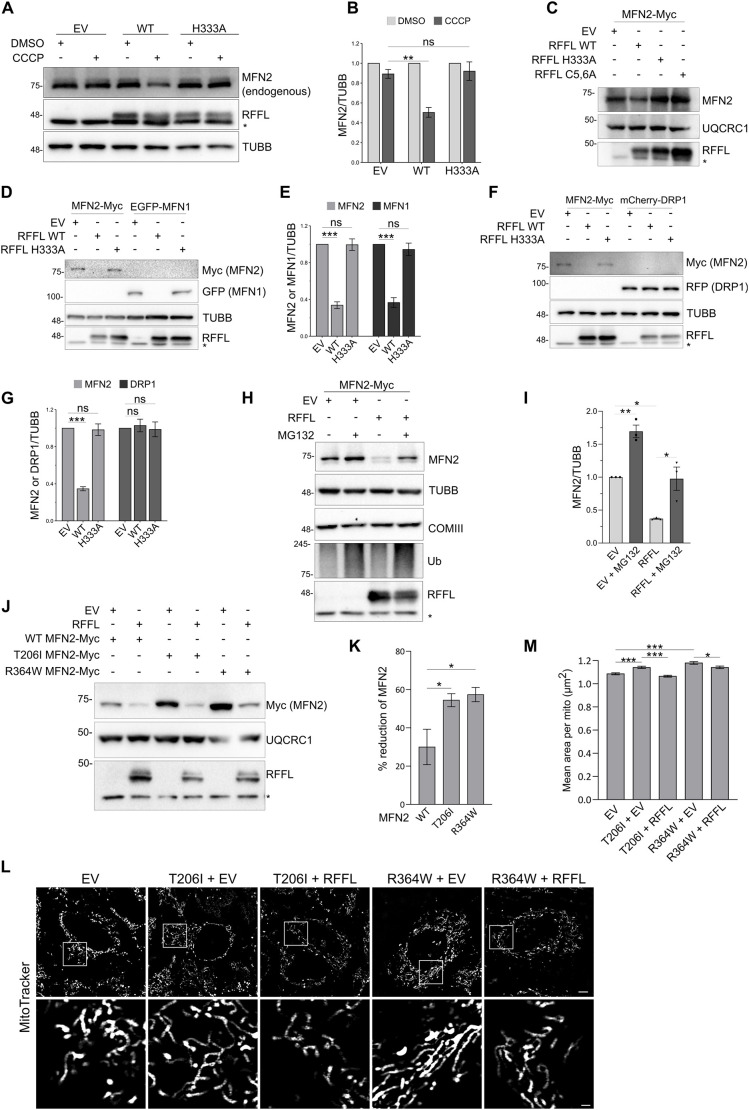
**RFFL degrades MFN2.** (A) Western blot showing levels of endogenous MFN2 in the lysates of A549 cells stably expressing EV or RFFL variants treated with DMSO or 20 μM CCCP for 45 min. * represents a non-specific band recognized by anti-RFFL antibodies. (B) Graph showing the ratio of band intensity of MFN2 to tubulin for experiments as in A (mean±s.d.) from three independent experiments. Values were normalized to DMSO value of each cell. Adjusted *P*-values for EV versus WT is 0.0011. (C) HEK293T cells were transfected with RFFL variants and MFN2–Myc as indicated. Cell extracts were prepared, and lysates were immunoblotted using indicated antibodies. * represents a non-specific band recognized by anti-RFFL antibodies. Blot shown representative of three experimental repeats. (D) HEK293T cells were transfected with indicated constructs and harvested after 48 h of transfection. * represents a non-specific band recognized by anti-RFFL antibodies. (E) Quantification of experiments as in D showing band intensities of MFN1 and MFN2 normalized to TUBB (mean±s.d.) from three independent experiments. EV ratio is considered as 1. (F) HEK293T cells were transfected with indicated constructs and harvested after 48 h of transfection. * represents a non-specific band recognized by anti-RFFL antibodies. (G) Quantification of experiments as in F showing band intensities of MFN2 or DRP1 normalized to TUBB (mean±s.d.) from three independent experiments. EV ratio is considered as 1. (H) HEK293T cells were transfected with indicated constructs, and the cell lysates were subjected to immunoblotting using indicated antibodies. Cells were harvested after 48 h of transfection with or without MG132 (10 μM) for 12 h. * represent a non-specific band recognized by anti-RFFL antibodies. (I) Quantification of experiments as in H where band intensity MFN2 normalized to tubulin band intensity was plotted (mean±s.d.) from three independent experiments. Adjusted *P*-values for EV versus EV+MG132 is 0.0072, EV versus RFFL is 0.0131 and RFFL versus RFFL+MG132 is 0.0164. (J) HEK293T cells were transfected with indicated constructs. Lysates were immunoblotted using indicated antibodies. * represents a non-specific band recognized by anti-RFFL antibodies. (K) Quantification of experiments as in J. Intensities of bands were measured, and percentage reduction of MFN2 was presented after normalization with UQCRC1. The error bar represents mean±s.d from three independent experiments. *P*-values for MFN2 WT versus T206I is 0.0464 and WT versus R364W is 0.0272. (L) HeLa cells were transfected with EV or untagged RFFL and myc-tagged MFN2 mutants. Mitochondria were stained with MitoTracker Red CMXRos and confocal live imaging was performed. Representative images are shown here, with contrast adjusted uniformly for better visibility. Post-imaging, cells were harvested and expression levels of transfected constructs were checked. Scale bars: 5 µm (main images); 1 µm (insets). (M) Quantification of experiments as in L. Field images were randomly captured in each condition, and the average area of a mitochondrion in a cell was calculated. Quantification was done from five independent experiments with a minimum of 30 cells per experimental condition. The error bar represents mean±s.e.m. *P*-values for R364W+EV versus R364W+RFFL is 0.0342. For all panels, *P<0.05; **P<0.01; ***P<0.001; ns, not significant. Statistical significances were calculated with a two-tailed unpaired *t*-test with Bonferroni's correction.

Given that we found direct interactions between RFFL and MFN2, which leads to the ubiquitylation of MFN2 *in vitro*, we looked at whether this ubiquitylation leads to the degradation of MFN2. We looked at the endogenous MFN2 in the lysate of A549 cells stably expressing EV or RFFL variants treated with DMSO or CCCP. A reduction in endogenous MFN2 was noted in cells stably expressing RFFL WT compared to EV upon CCCP treatment (Fig. 5A,B). However, this reduction was not observed in the presence of ligase inactive RFFL (H333A) (Fig. 5A,B). Given that endocytic vesicle-associated RFFL appeared to target MFN2 (Fig. 2D; Movies 5, 6), we wondered whether the expression of the endosomal-association-defective RFFL variant (RFFL C5, 6A) could also target MFN2 for degradation (Ravindran et al., 2022). When the RFFL C5, 6A variant was expressed along with MFN2–Myc, unlike WT, variant (C5, 6A) showed no effect on MFN2 protein level (Fig. 5C). As the C5, 6A variant of RFFL could still interact with MFN2 (Fig. 3D), the data suggests that the endosome association of RFFL is important for the degradation of MFN2 by RFFL, further highlighting the importance of endosome–mitochondria contacts. As expected, the reduction in MFN2 levels was not observed with the inactive RFFL E3 mutant (H333A), further indicating the involvement of the ubiquitin ligase activity of RFFL in the reduction of MFN2 protein (Fig. 5C). Given that we observed a robust reduction in MFN2 levels by RFFL under overexpression conditions, we next investigated whether RFFL targets other proteins involved in mitochondrial fusion and fission. Specifically, we examined MFN1, a key mitochondrial fusion protein closely related to MFN2 and ranked second in our MLM score-based ranking (Fig. 2A), as well as DRP1 (also known as DNM1L), a mitochondrial fission factor, to assess the effect of RFFL (Dorn, 2019). For this, we used MFN2 as positive control and co-transfected EGFP–MFN1 or mCherry–DRP1 along with RFFL in HEK293T cells. Not surprisingly, RFFL also targeted MFN1 for degradation to a similar extent as MFN2 (Fig. 5D,E). However, RFFL expression was unable to reduce levels of DRP1 protein, suggesting the specificity of RFFL towards MFN1 and MFN2 (Fig. 5F,G).

We then co-transfected HEK293T cells with MFN2–Myc along with EV or untagged RFFL WT and treated the cells with proteasome inhibitor MG132 (Tsubuki et al., 1996) to assess whether the reduction in MFN2 was the result of proteasomal degradation. Upon MG132 treatment, the protein levels of MFN2–Myc moderately increased not only in EV control but also in cells expressing RFFL, suggesting the contribution of RFFL to MFN2 degradation by proteasomal machinery (Fig. 5H,I). Because RFFL appears to target MFN2, and lack of RFFL results in hyperfused mitochondria, we wondered about the effect of RFFL on MFN2 variants that are known to cause hyperfusion of mitochondria. Two MFN2 mutations have been reported to be associated with CMT2A, namely, MFN2 R364W and T206I, and are known to cause mitochondrial hyperfusion (Das et al., 2022a,b; Das et al., 2024; El Fissi et al., 2018; Stuppia et al., 2015; Verhoeven et al., 2006). Interestingly, the pathological and hyperfusion mutant variants of MFN2 showed increased susceptibility to degradation by RFFL than WT (Fig. 5J,K). Given that these MFN2 mutants are reported to cause hyperfusion of mitochondria, we wondered whether RFFL could rescue the effect of the CMT2A-associated mutants. We overexpressed these mutants in HeLa cells and observed mitochondrial morphology using confocal microscopy. As reported, we saw hyperfused mitochondria in the presence of T206I and R364W mutants of MFN2. Importantly, co-transfection with RFFL decreased this hyperfusion, bringing back the mitochondria morphology to normal (EV) levels (Fig. 5L,M; Fig. S3B).

### RFFL affects lipid homeostasis

**Fig. 6.**
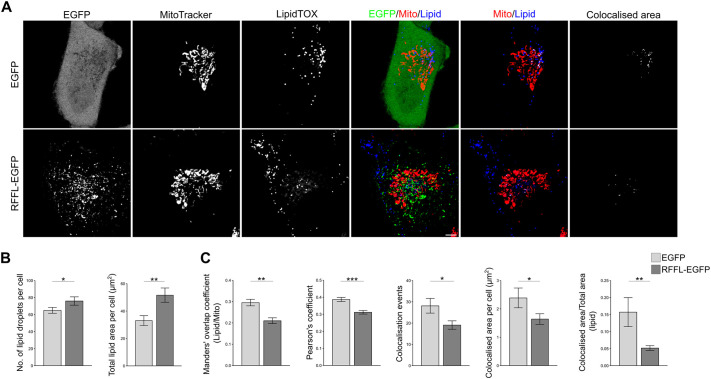
**Lipid homeostasis is affected by RFFL.** (A) A549 stable cells expressing EGFP or RFFL–EGFP were stained with MitoTracker™ Red CMXRos and HCS LipidTOX™ Deep Red Neutral Lipid Stain, and confocal live imaging was performed. Figure contrasts are adjusted uniformly for better visibility. Scale bar: 5 µm. (B,C) Quantification based on A. A minimum 30 cells for each of three biological replicates was used for quantification and mean±s.e.m is plotted. Statistical significance was calculated using two-tailed unpaired *t*-test. *P*-values for Manders' is 0.0014. Number of LDs, 0.0319; total lipid area, 0.0036; colocalised events, 0.0135, colocalized area, 0.0286; colocalized area of lipid to total lipid area, 0.0086. *P<0.05; **P<0.01; ***P<0.001.

Given that endosome–mitochondria association facilitates lipid exchange (Ouyang et al., 2023), we investigated the effect of RFFL expression on the accumulation of lipid droplets (LDs), considered as energy storage units in cells (Enkler and Spang, 2024; Kumar et al., 2024; Mallick et al., 2024). For this, we stained A549 cells stably expressing RFFL–EGFP or EGFP alone with HCS LipidTOX Deep Red, a dye that stains neutral lipids. Confocal images of these cells showed differences in lipid staining between the cells with or without RFFL overexpression (Fig. 6A). Quantification of LDs showed that there were more LDs in RFFL-expressing cells with a substantial increase in LDs area than in control cells (Fig. 6B). Interestingly, the colocalization between LDs and mitochondria (stained with MitoTracker Red CMXRos), measured by different parameters, were also found to be less in RFFL-expressing cells (Fig. 6C). These results are consistent with observations reported earlier in cells with reduced MFN2 (Cai et al., 2024; Hu et al., 2024). These results indicate a hitherto unknown role for RFFL in lipid homeostasis.

## DISCUSSION

Cellular homeostasis is maintained by mitochondrial dynamics established through numerous contacts with diverse organelles like the ER, endosomes and lysosomes, in addition to mitochondria themselves. These continuous crosstalks affect mitochondrial morphology and diverse signaling like lipid homeostasis, synaptic plasticity, cell death and autophagy by aiding the exchange of mtDNA, ions, lipids and metabolites (Collier et al., 2023; Harper et al., 2020). Recently, research on mitochondria–endosome crosstalk (MECS) has been gaining momentum (Müntjes et al., 2021; Todkar et al., 2019). MECS has been reported to aid iron and cholesterol transfer between endosomes and mitochondria (Charman et al., 2010; Das et al., 2016; Hamdi et al., 2016; Nara et al., 2023; Satoh et al., 2023; Sheftel et al., 2007; Wang et al., 2020). Here, we show that RFFL endosomes come into close contact with mitochondria and alter mitochondrial morphology by specifically targeting outer mitochondrial membrane (OMM) proteins involved in mitochondrial fusion (MFN1 and MFN2) but not fission-associated protein DRP1. Moreover, we demonstrate that exogenous expression of RFFL results in an increased lipid content.

Lipid droplets (LDs) are primarily made up of neutral lipids, such as triglycerides and cholesterol. They form highly dynamic connections with various organelles, including the ER, endosomes and mitochondria (Enkler and Spang, 2024; Peng et al., 2025). Although many aspects of lipid–organelle crosstalk are not yet understood, LDs are at the center of lipid and energy homeostasis in cells, and it is increasingly believed that their dynamic association with organelle are coupled to the cycles of lipid droplet expansion and shrinkage (Enkler and Spang, 2024; Zadoorian et al., 2023). Our findings that RFFL expression leads to not only a reduction in colocalization of LDs with mitochondria but an increase in LD numbers and area suggest that these changes could be the consequence of RFFL-mediated changes in MFN2 protein levels. MFN2 is reported to connect mitochondria to LDs via its association with perilipin 1 (PLIN1), a member of the perilipin family on LDs, and suppress lipid accumulation in cells (Boutant et al., 2017; Cai et al., 2024; Mahdaviani et al., 2017). Interestingly, PRKN is also known to be involved in lipid remodeling during mitophagy (Lin et al., 2022; Tang et al., 2023). Combined with our earlier observation of delayed PRKN recruitment to damaged mitochondria in RFFL KO cells, this study establishes a novel role for RFFL and endosomes in mitochondrial dynamics, mitophagy and lipid homeostasis.

MFN2 is known to be ubiquitylated in cells upon mitochondrial depolarization (Gegg et al., 2010; McLelland et al., 2018). Although several E3 ligases, namely MARCHF5 and MUL1 (mitochondria), PRKN, RBCK1 and HUWE1 (cytosolic), and AMFR (ER) are reported to contribute to MFN2 ubiquitylation, none of these E3 ligases has been shown to directly modify MFN2 nor associate with endosomes. In this study, we demonstrate that RFFL interacts and ubiquitylates MFN2 *in vivo* and *in vitro*, and loss of RFFL results in reduced MFN2 burst upon CCCP treatment. These results clearly indicate that RFFL is an E3 for MFN2. Furthermore, for the first time, we demonstrate the ubiquitylation of MFN2 by any E3 using recombinant proteins. Interestingly, unlike UBE2D1 and UBE2D2, E2s that facilitate ubiquitylation of both MFN2 and autoubiquitylation of RFFL, UBE2W modifies MFN2 but not RFFL, suggesting differential RFFL activity with different E2s. These differences can be of significance in the physiological context, given the reported diverse tissue-specific roles for MFN2 (Chandhok et al., 2018). MFN2 splice variants dubbed ER mitofusin 2 (ERMIN2) and ER mitofusin 2 tether (ERMIT2) have been reported. The former is involved in shaping the ER, and the latter in ER–mitochondria tethering (Naón et al., 2023). It will be interesting to explore the effect of RFFL on these isoforms. Given that no endosomal-associated E3 ligase is known to control MFN2 turnover and mitochondrial morphology so far, identifying RFFL as an E3 ligase for MFN2 opens a new avenue to explore endosome mitochondria crosstalk. Consistent with such a possibility, a recent unbiased analysis of the endogenous interactome of MFNs has identified a number of endosomal-associated proteins (Gordaliza-Alaguero et al., 2025).

Given that our data demonstrate that RFFL binds and ubiquitylates the cytosol facing N-terminus of MFN2 (1-357 amino acids, containing GTPase domain) like the full-length MFN2, it raises the possibility that under physiological conditions, RFFL can influence the GTPase activity and subsequent MFN2 dimerization and membrane fusion. It is interesting to note that the majority of mutations of MFN2 reported in individuals with CMT2A are concentrated in the N terminus of MFN2 (GTPase domain) (Cartoni and Martinou, 2009; Filadi et al., 2018; Züchner et al., 2004). Exogenous expression of the CMT2A pathogenic variants of MFN2 (T206I and R364W) are known to promote hyperfusion of mitochondria, reportedly by stabilizing the mutated protein level (Das et al., 2022b, 2024). Importantly, we show that co-expression with RFFL not only reduced the protein levels of both the pathogenic variants but also rescued the hyperfusion phenotype of mitochondria. Restoring impaired mitochondrial dynamics in CMT2A disease is an active avenue of research (Franco et al., 2016; Rocha et al., 2018). Hence, the observation that there is a reduction in the levels of pathogenic MFN2 variants mediated by RFFL can open up a potential therapeutic option by modulating RFFL activity. Interestingly, mutations in not only MFN2 but also endosomal Rab7 proteins have been implicated in the development of Charcot–Marie–Tooth neuropathy diseases (Meggouh et al., 2006; Ponomareva et al., 2016). It is also possible that the RFFL–MFN2 crosstalk affects signaling pathways other than mitochondrial dynamics. For example, RFFL positively regulates cell migration and proliferation (Dong et al., 2013; Gan et al., 2012, 2013; Sharma et al., 2024), with MFN2 controlling negatively (Chen et al., 2004, 2014; Guo et al., 2007; Shen et al., 2007), making it another avenue for exploration.

Overall, the findings of this study provide novel insights into the intricate interplay between RFFL, mitochondrial dynamics and the regulation of MFN2 turnover. By elucidating the role of RFFL in modulating mitochondrial morphology and its direct interaction with MFN2, this research lays the groundwork for further exploration of the underlying molecular mechanisms and the potential implications for cellular homeostasis and disease pathogenesis. These findings have the potential to pave the way for the development of novel therapeutic strategies targeting RFFL-mediated regulation of mitochondrial dynamics and lipid homeostasis in various pathological conditions associated with mitochondrial dysfunction.

## MATERIALS AND METHODS

### Cell culture

A549 (ATCC, #CRM-CCL-185), HeLa (ATCC, #CRM-CCL-2) and HEK293T cells (ATCC, #CRL-3216) were grown in Dulbecco's modified Eagle's medium (Thermo Fisher Scientific, 10569-010) supplemented with 10% heat-inactivated fetal bovine serum (Thermo Fisher Scientific, 10270-106) and 100 U/ml penicillin as well as 100 µg/ml streptomycin (Thermo Fisher Scientific, 15140122) at 37°C in a humidified chamber with 5% CO_2_ as described previously (Ravindran et al., 2022).

### Transfection and generation of stable and KO cells

Transfection of cells with plasmid DNA was undertaken using Lipofectamine 3000 reagent (Thermo Fisher Scientific, L3000-015) according to the manufacturer's instructions. The total amount of DNA in all wells was kept constant using empty vector DNA in all experiments. Cells stably expressing different constructs were generated as described previously (Ravindran et al., 2022). A549 RFFL KO cells were generated as described previously (Ravindran et al., 2022).

### Plasmids and antibodies

Human CARP2 cDNA (NP_001017368.1) and its variants were cloned to pMYs-IP (Kitamura et al., 2003) for stable cell generation (Ravindran et al., 2022). RFFL variants were cloned into pGEX-4T-2 (GE Healthcare Life Sciences, #27458101) for bacterial expression and purification and pcDNA 3.1 vector (Invitrogen, #V790-20) for transfection in mammalian cells. MFN2–YFP was a generous gift from Atsushi Tanaka (Biochemistry Section, National Institutes of Health, USA; Tanaka et al., 2010). MFN2 cDNA (NP_001121132.1) was cloned into piRFP670-N1 for imaging studies. piRFP670-N1 was Addgene plasmid #45457, deposited by Vladislav Verkhusha (Shcherbakova and Verkhusha, 2013). WT MFN2–Myc, T206I MFN2–Myc and R364W MFN2–Myc constructs were kind gifts from Prof. Oishee Chakrabarti (Biophysics and Structural Genomics Division, Saha Institute of Nuclear Physics, India; Das et al., 2022a,b, 2024). pERS28a was a kind gift from Dr Ramanathan Natesh (School of Biology, Indian Institute of Science Education and Research Thiruvananthapuram, India; Maddi and Natesh, 2021). MFN2 was cloned to the pERS28a or pET-28a vector (EMD Biosciences, #69864-3) for expression and purification from bacteria. YFP–Parkin was Addgene plasmid #23955, deposited by Richard Youle (Narendra et al., 2008). pRK5-HA-Ubiquitin-WT was Addgene plasmid #17608, deposited by Ted Dawson (Lim et al., 2005). pRK5-HA-Ub-K0 was a kind gift from Prof. Subramaniam Ganesh (Biological Sciences & Bioengineering, Indian Institute of Technology Kanpur, India). mCherry–DRP1 was Addgene plasmid #49152, deposited by Gia Voeltz (Friedman et al., 2011). EGFP-MFN1 was generated by cloning MFN1 cDNA into pEGFP-C1 (Mukherjee and Chakrabarti, 2016). Ub cDNA (untagged or fused with GST) was subcloned to pET-3A (Novagen, #69418) for protein purification. All plasmids contain cDNA of human origin.

Antibodies against the following proteins were used in this study; TOMM20 (1:1000, Santa Cruz Biotechnology, sc-17764), MFN2 (1:2000, Proteintech, 12186-1-AP), HA (1:2000, Santa Cruz Biotechnology, sc-7392), ubiquitin (1:2000, Santa Cruz Biotechnology, sc-8017), GST (1:2000, Sigma-Aldrich, G7781), Myc (1:500, DSHB, 9E 10), RFFL (Ravindran et al., 2022), GAPDH (1:2000, Abgenex, 10-10011), tubulin (1:5000, Sigma-Aldrich, T6199), UQCRC1 (1:2000, Invitrogen, 16D10AD9AH5), GFP (1:1000, Mouse Living Colors Clontech, 632375 or SCBT, sc-9996), RFP (1:2000, ChromoTek, 5f8-100) and parkin (1:2000, Santa Cruz Biotechnology, sc-32282). Anti-mouse-IgG HRP (1:5000, Invitrogen, 61-6520/Jackson ImmunoResearch, 115-035-174) or anti-rabbit-IgG HRP (1:5000, Invitrogen, 65-6120/Jackson ImmunoResearch, 211-032-171) were used to visualize the immunoblots with chemiluminescence.

### Reagents

For mitochondrial staining, cells were incubated with 10 nM MitoTracker Red CMXRos (M7512, Gibco-Thermo Fisher Scientific) for 30 min in medium. For lipid staining, HCS LipidTOX™ Deep Red Neutral Lipid Stain (H34477, Invitrogen, Carlsbad, CA, USA) was added at 1:5000 dilution for 25 min after MitoTracker Red CMXRos staining. DAPI (D8417, Sigma-Aldrich) staining was performed as described previously (Ravindran et al., 2022). The following chemicals were used: MG132 (M7449, SigmaAldrich), Bafilomycin A1 (19-148, Merck, Darmstadt, Germany), and CCCP (C-2759, Sigma-Aldrich).

### Immunoprecipitation and western blotting

Immunoprecipitation experiments (both native and denaturation conditions) were performed as described previously (Ravindran et al., 2022). Western blotting experiments were done using standard protocols. To assess MFN2–Myc degradation, HEK293T cells were co-transfected with 400 ng of untagged RFFL and 1000 ng MFN2–Myc constructs in a 35 mm dish. After 36–48 h of transfection, cells were harvested (500 ***g*** for 2 min) and suspended in SNTBS buffer (50 mM Tris-HCl pH 7, 150 mM NaCl, 2% SDS, 1% NP-40) with 1 mM phenylmethylsulfonyl fluoride (Sigma-Aldrich, P7626) and 1× protease inhibitor cocktail (Sigma-Aldrich, P8340). The extracts were sonicated (Sha et al., 2019) and incubated at 37°C for 30 min before loading on SDS-PAGE gels. Unless otherwise specified, for MFN2 detection, samples were heated at 37°C for 30 min with Laemmli buffer (Schägger, 2006; Yamano et al., 2020). Quantification of bands was done from western blot images taken without a saturating signal.

### Electron microscopy imaging and analysis

Cells were seeded in a T75 flask for 24 h and collected at ∼90% confluency. Cells were washed with 100 mM phosphate buffer and fixed with fixative (2.5% glutaraldehyde plus 2.0% paraformaldehyde in 0.1 M phosphate buffer) for 2 h at room temperature. Cells were scrapped after fixation and washed three times with phosphate buffer for 15 min each and post-fixed with 1% osmium tetroxide for 1 h at 4°C. After fixation, cells were dehydrated in a graded series of ethanol (30%, 50%, 60%, 70%, 80%, 90% and 100%). These samples were infiltrated with toluene/resin and embedded in Epon 812 resin. The resin-embedded samples were polymerized at 65°C for 48 h. The ultrathin sections of 70 nm were cut using Leica UC7 ultramicrotome and mounted on the carbon-coated 200 mesh copper grid. The sections were heavy metal stained using uranyl acetate and lead citrate, then viewed under a transmission electron microscope Talos F200S (Thermo Fisher Scientific).

Individual mitochondria in each cell were marked as regions of interest (ROI) for quantification, and images were calibrated. Then, the area and perimeter were measured using ImageJ software (ImageJ 1.53t) (Schneider et al., 2012). Feret's diameter is defined as the longest distance between any two points along the selection boundary and was also measured using ImageJ. Mitochondria marking and measurement were done by a person who was unaware of the experimental conditions.

### Immunocytochemistry and confocal microscopy

For fixed-cell imaging, cells were grown on coverslips and immunostained as described previously (Ravindran et al., 2022). Images were acquired in Zeiss LSM 880 confocal microscope (Carl Zeiss, Germany) with either a Plan-Apochromatic 63×/1.4 or a 100×/1.4 oil immersion objective. For live-cell imaging, cells were cultured in a glass bottom dish (Cell Vis, D35-20-1.5-N) and imaged using the FV3000 confocal microscope (Olympus, Japan) with an UPLXAPO 60×/1.42 oil immersion objective lens. The FV3000 system had an attached STX stage top incubator (TOKAI HIT, Japan), humidified with 5% CO_2_ at 37°C. 2D deconvolution was performed on all time-lapse movies for better visibility using Olympus cellSens Dimension Desktop 3.2. Image contrast was adjusted using the lookup table for better visibility of representative Figures. For colocalization analysis, BIO-jacop plugin was used after auto thresholding (https://github.com/BIOP/ijp-jacop-b; Bolte and Cordelières, 2006). Lipid-mitochondria analysis was performed using the analyze particle function and colocalization plugin after auto thresholding (https://imagej.net/ij/plugins/colocalization.html). All quantifications were performed from raw confocal images unless otherwise indicated.

### Mitochondria morphology imaging and analysis

A549 cells were fixed and immunostained against endogenous TOMM20 as described above. Fixed cells were imaged using Zeiss LSM 880 with Plan-Apochromat 100×/1.40 oil objective. The pinhole was set to 0.75 airy unit (AU). Around 20–35 *Z*-sections were taken for each image with a 250 nm step size. Bidirectional scanning was enabled for fast capture. These images were deconvoluted using the Fiji (ImageJ 1.53t) plugin theoretical PSF generator (Kirshner et al., 2013) and deconvolution lab2 (Version 2.1.2). PSF was calculated using the Born and Wolf 3D Optical Model with the best accuracy. Deconvolution was performed using the Richardson–Lucy algorithm with ten iterations. Deconvoluted images were saved, calibrated and converted into 8-bit images. These images were thresholded first using mitochondria analyzer plugin (Chaudhry et al., 2020) with pre-processing commands subtract background (rolling 1.25), sigma filter plus (radius 0.6), enhance local contrast (max slope 1.4), adjust gamma (0.9), local threshold (weighted mean), C value 3.5, block size 1.25 µm and post-processing commands, Despeckle, Remove outliers (radius 0.6 pixels) and Fill 3D holes commands were enabled. Analysis was performed on these thresholded images after drawing a region of interest on single cells and clearing out the rest of the field. The 3D view was generated using a 3D viewer plugin available in Fiji (Schindelin et al., 2012).

For calculating the average area of mitochondria per cell, live imaging was performed and 2D images of mitochondria stained with MitoTracker Red CMXRos were captured. The pinhole was set to 0.75 airy units (AU). These images were deconvoluted using Olympus cellSens Dimension Desktop 3.2. The analyze particle function in Fiji software was used to remove pixels less than 0.4 µm. Then, the mitochondria analyzer plugin (Chaudhry et al., 2020) was used as described above to segment mitochondria, followed by area measurement.

Manual measurement of mitochondrial length and width was measured as described previously (Lam et al., 2021). Briefly, for mitochondrial length, the freehand line tool in ImageJ was used. Ten random positions per cell where mitochondria were not clustered were measured using this tool. Similarly, the width of the mitochondria was measured using the straight line tool. The percentage of cells with fragmented, normal and interconnected mitochondria was calculated based on the length. Measurements were done by a person who was unaware of the experimental conditions. Mitochondria with a length of <2 μm considered as fragmented, 2–5 μm as normal (tubular), and >5 μm as interconnected (filamentous or hyperfused) (Das et al., 2022a).

### *In vitro* experiments and recombinant protein purification

For *in vitro* interaction experiments, His-tagged MFN2 constructs and GST-tagged RFFL were co-transformed in Arctic Express *E. coli* cells, and transformants with both constructs were selected on agar plates with antibiotics: 10 μg/ml gentamicin (Sigma-Aldrich, #G1272), 20 μg/ml kanamycin (Himedia, CMS7172) and 100 μg/ml ampicillin (Himedia, #MB104). The bacterial culture was grown from a single colony at 37°C with rotation at 220 r.p.m. until the optical density at 600 nm (OD600) reached 0.4. Then 150 µM of IPTG was added, and the culture was shifted to 10°C for 24 h at 220 r.p.m. The culture was pelleted (3000 ***g*** for 10 min), and the cell pellet was lysed in buffer A (50 mM NaH_2_PO_4_ pH 8.0, 15 mM 2-mercaptoethanol, 500 mM NaCl, 25 mM trehalose, 5 mM MgCl_2_, 0.025 mM MnCl_2_, 5% glycerol, 0.5% Triton-X, 0.714 mg/ml lysozyme, 2 mM PMSF and 1× protease inhibitor (Sigma-Aldrich, cOmplete Mini Protease Inhibitor Cocktail). Cells were then lysed by sonication and centrifuged at 18,000 ***g*** for 10 mins at 4°C. The supernatant was collected and incubated with pre-washed Glutathione beads (GE Healthcare, Glutathione Sepharose 4 Fast Flow) for 4 h at 4°C. Beads were then washed three times with buffer B (50 mM NaH_2_PO_4_ pH 8.0, 15 mM 2-mercaptoethanol, 500 mM NaCl, 5 mM MgCl_2_ and 5% glycerol) and then resuspended in 2X Laemmli buffer and heated for 30 min at 37°C.

For *in vitro* ubiquitylation experiments, RFFL and MFN2 were co-expressed, and GST pulldown was performed as described above. Then GST-bound Glutathione beads were washed in Buffer C (50 mM Tris-HCl pH 7.4 and 5 mM MgCl_2_) and re-suspended in 30 µl Buffer C with an E1 (10 nM UBA1; R&D Systems, E-304) and E2 (0.2 µM; see below) enzyme, Ub (0.2 ug), 2 mM DTT and 2 mM ATP. The samples were incubated at 30°C for 90 min with rotation at 600 r.p.m., and the reaction was stopped by adding the final 2× Laemmli buffer and heated for 30 min at 37°C. The following E2 enzymes were used in this study: UBE2W (E2-725) UBE2H (K-980B) UBE2R1 (K-980B) UBE2D1 (K-980B) UBE2D2 (K-980B) UBE2E1 (K-980B) UBE2E3 (K-980B) UBE2L3 (K-980B) UBE2C (K-980B) UBE2N/Uev1a (E2-664) UBE2G2 (E2-680), from R&D Systems, USA. If the E2 is not specified, UBE2D1 was used. Me-Ub was from R&D Systems, USA (U-501).

Recombinant ubiquitin was produced through overexpression in *E. coli* BL21 (DE3) competent cells. A singular colony was introduced into LB broth supplemented with 100 µg/ml ampicillin and cultured at 37°C for 12 h. The resulting primary culture was transferred to 1 liter of LB broth containing 100 µg/ml ampicillin and maintained at 37°C until the OD600 reached was 0.4–0.6. Subsequently, cells were induced with 1 mM IPTG and further incubated at 37°C for 5 h. Upon completion of the induction period, the culture was centrifuged at 9820 ***g*** for 10 min at 4°C and stored at −80°C. The thawed cell pellet was suspended in a 25 ml lysis buffer (50 mM sodium acetate and 5 mM EDTA, pH 5.1, 2.87 mM PMSF, and one cOmplete, mini, EDTA free protease inhibitor cocktail tablet; Roche) and subjected to sonication for 3 min (pulse: 20 s on, 30 s off; amplitude, 55%) on ice. The lysed cells were then centrifuged at 101,633 ***g*** for 90 min at 4°C. The resulting supernatant was applied to an SP Sepharose column, pre-equilibrated with sodium acetate buffer (matching the lysis buffer but without PMSF and protease inhibitor), and left overnight in a cold room. After washing the beads with sodium acetate buffer, protein elution was achieved using a sodium chloride gradient ranging from 10 to 250 mM in sodium acetate buffer. The eluted fractions, assessed by 15% SDS-PAGE and Coomassie staining, were pooled based on comparable purity. Subsequent dialysis (50 mM Tris-HCl pH 7.4, 5 mM MgCl_2_ and 2 mM DTT in deionized water) was conducted for 8 h at 4°C, with buffer changes occurring at four-hour intervals. The dialyzed protein was concentrated further using Amicon Ultra-4 centrifugal filters with a 3 K regenerated cellulose membrane. Protein concentration was determined, and aliquots were preserved at −80°C.

### Protein–protein interaction prediction

To predict how likely a pair of proteins bind to each other, we used protein language model ESM-2 (Lin et al., 2023). The list of prey proteins was concatenated in pairs with bait protein, and Masked Language Model loss (MLM loss) was calculated using the masked language modeling capabilities of ESM-2 and randomly mask residues (Lupo et al., 2024). We used esm2_t30_150M_UR50D with 150 million parameters for this study.

### Statistical analysis and software

Data were collected from at least three independent experiments. Significance levels were set at 0.05, and are marked as *P<0.05, **P<0.01, and ***P<0.001. All statistical analyses were performed in GraphPad Prism (Version 8.3.1). All graphs were generated in GraphPad Prism. Figures were arranged using Inkscape 1.2 (https://inkscape.org/). For protein domain illustrations, we used DOG 2.0 and modified (Ren et al., 2009).

## Supplementary Material



10.1242/joces.263830_sup1 Supplementary information

## References

[JCS263830C1] Araki, K., Kawamura, M., Suzuki, T., Matsuda, N., Kanbe, D., Ishii, K., Ichikawa, T., Kumanishi, T., Chiba, T., Tanaka, K. et al. (2003). A palmitoylated RING finger ubiquitin ligase and its homologue in the brain membranes. *J. Neurochem.* 86, 749-762. 10.1046/j.1471-4159.2003.01875.x 12859687

[JCS263830C2] Bolte, S. and Cordelières, F. P. (2006). A guided tour into subcellular colocalization analysis in light microscopy. *J. Microsc.* 224, 213-232. 10.1111/j.1365-2818.2006.01706.x 17210054

[JCS263830C3] Boutant, M., Kulkarni, S. S., Joffraud, M., Ratajczak, J., Valera–Alberni, M., Combe, R., Zorzano, A. and Cantó, C. (2017). Mfn2 is critical for brown adipose tissue thermogenic function. *EMBO J.* 36, 1543-1558. 10.15252/embj.201694914 28348166 PMC5452040

[JCS263830C4] Cai, Z., Luo, W., Wang, H., Zhu, R., Yuan, Y., Zhan, X., Xie, M., Zhuang, H., Chen, H., Xu, Y. et al. (2024). MFN2 suppresses the accumulation of lipid droplets and the progression of clear cell renal cell carcinoma. *Cancer Sci.* 115, 1791-1807. 10.1111/cas.16151 38480904 PMC11145141

[JCS263830C5] Cartoni, R. and Martinou, J.-C. (2009). Role of mitofusin 2 mutations in the physiopathology of Charcot–Marie–Tooth disease type 2A. *Exp. Neurol.* 218, 268-273. 10.1016/j.expneurol.2009.05.003 19427854

[JCS263830C6] Cartoni, R., Arnaud, E., Médard, J.-J., Poirot, O., Courvoisier, D. S., Chrast, R. and Martinou, J.-C. (2010). Expression of mitofusin 2(R94Q) in a transgenic mouse leads to Charcot-Marie-Tooth neuropathy type 2A. *Brain J. Neurol.* 133, 1460-1469. 10.1093/brain/awq082 20418531

[JCS263830C7] Chandhok, G., Lazarou, M. and Neumann, B. (2018). Structure, function, and regulation of mitofusin–2 in health and disease. *Biol. Rev. Camb. Philos. Soc.* 93, 933-949. 10.1111/brv.12378 29068134 PMC6446723

[JCS263830C8] Charman, M., Kennedy, B. E., Osborne, N. and Karten, B. (2010). MLN64 mediates egress of cholesterol from endosomes to mitochondria in the absence of functional Niemann-Pick Type C1 protein. *J. Lipid Res.* 51, 1023-1034. 10.1194/jlr.M002345 19965586 PMC2853429

[JCS263830C9] Chaudhry, A., Shi, R. and Luciani, D. S. (2020). A pipeline for multidimensional confocal analysis of mitochondrial morphology, function, and dynamics in pancreatic β-cells. *Am. J. Physiol. Endocrinol. Metab.* 318, E87-E101. 10.1152/ajpendo.00457.2019 31846372 PMC7052579

[JCS263830C10] Chen, H. and Chan, D. C. (2005). Emerging functions of mammalian mitochondrial fusion and fission. *Hum. Mol. Genet.* 14, R283-R289. 10.1093/hmg/ddi270 16244327

[JCS263830C11] Chen, Y. and Dorn, G. W. (2013). PINK1-phosphorylated mitofusin 2 is a parkin receptor for culling damaged mitochondria. *Science* 340, 471-475. 10.1126/science.1231031 23620051 PMC3774525

[JCS263830C12] Chen, H., Detmer, S. A., Ewald, A. J., Griffin, E. E., Fraser, S. E. and Chan, D. C. (2003). Mitofusins Mfn1 and Mfn2 coordinately regulate mitochondrial fusion and are essential for embryonic development. *J. Cell Biol.* 160, 189-200. 10.1083/jcb.200211046 12527753 PMC2172648

[JCS263830C13] Chen, K.-H., Guo, X., Ma, D., Guo, Y., Li, Q., Yang, D., Li, P., Qiu, X., Wen, S., Xiao, R.-P. et al. (2004). Dysregulation of HSG triggers vascular proliferative disorders. *Nat. Cell Biol.* 6, 872-883. 10.1038/ncb1161 15322553

[JCS263830C14] Chen, K.-H., Dasgupta, A., Ding, J., Indig, F. E., Ghosh, P. and Longo, D. L. (2014). Role of mitofusin 2 (Mfn2) in controlling cellular proliferation. *FASEB J.* 28, 382-394. 10.1096/fj.13-230037 24081906 PMC3868832

[JCS263830C15] Chen, W., Zhao, H. and Li, Y. (2023). Mitochondrial dynamics in health and disease: mechanisms and potential targets. *Signal Transduct. Target. Ther.* 8, 333. 10.1038/s41392-023-01547-9 37669960 PMC10480456

[JCS263830C16] Collier, J. J., Oláhová, M., McWilliams, T. G. and Taylor, R. W. (2023). Mitochondrial signalling and homeostasis: from cell biology to neurological disease. *Trends Neurosci.* 46, 137-152. 10.1016/j.tins.2022.12.001 36635110

[JCS263830C17] Cosentino, K. and García-Sáez, A. J. (2014). Mitochondrial alterations in apoptosis. *Chem. Phys. Lipids* 181, 62-75. 10.1016/j.chemphyslip.2014.04.001 24732580

[JCS263830C18] Coumailleau, F., Das, V., Alcover, A., Raposo, G., Vandormael-Pournin, S., Le Bras, S., Baldacci, P., Dautry-Varsat, A., Babinet, C. and Cohen-Tannoudji, M. (2004). Over-expression of Rififylin, a new RING finger and FYVE-like domain-containing protein, inhibits recycling from the endocytic recycling compartment. *Mol. Biol. Cell* 15, 4444-4456. 10.1091/mbc.e04-04-0274 15229288 PMC519139

[JCS263830C19] Das, A., Nag, S., Mason, A. B. and Barroso, M. M. (2016). Endosome–mitochondria interactions are modulated by iron release from transferrin. *J. Cell Biol.* 214, 831-845. 10.1083/jcb.201602069 27646275 PMC5037410

[JCS263830C20] Das, R., Das, S., Chakrabarti, S. and Chakrabarti, O. (2022a). CMT2A-linked mitochondrial hyperfusion-driving mutant MFN2 perturbs ER-mitochondrial associations and Ca^2+^ homeostasis. *Biol. Cell* 114, 309-319. 10.1111/boc.202100098 35924634

[JCS263830C21] Das, R., Kamal, I. M., Das, S., Chakrabarti, S. and Chakrabarti, O. (2022b). MITOL-mediated DRP1 ubiquitylation and degradation promotes mitochondrial hyperfusion in a CMT2A-linked MFN2 mutant. *J. Cell Sci.* 135, jcs257808. 10.1242/jcs.257808 34870686

[JCS263830C22] Das, R., Maity, S., Das, P., Kamal, I. M., Chakrabarti, S. and Chakrabarti, O. (2024). CMT2A-linked MFN2 mutation, T206I promotes mitochondrial hyperfusion and predisposes cells towards mitophagy. *Mitochondrion* 74, 101825. 10.1016/j.mito.2023.101825 38092249

[JCS263830C23] Di Rita, A., Peschiaroli, A., D'Acunzo, P., Strobbe, D., Hu, Z., Gruber, J., Nygaard, M., Lambrughi, M., Melino, G., Papaleo, E. et al. (2018). HUWE1 E3 ligase promotes PINK1/PARKIN-independent mitophagy by regulating AMBRA1 activation via IKKα. *Nat. Commun.* 9, 3755. 10.1038/s41467-018-05722-3 30217973 PMC6138665

[JCS263830C24] Dong, Y., Zhao, J., Wu, C.-W., Zhang, L., Liu, X., Kang, W., Leung, W.-W., Zhang, N., Chan, F. K. L., Sung, J. J. Y. et al. (2013). Tumor suppressor functions of miR-133a in colorectal cancer. *Mol. Cancer Res.* 11, 1051-1060. 10.1158/1541-7786.MCR-13-0061 23723074

[JCS263830C25] Dorn, G. W. (2019). Evolving concepts of mitochondrial dynamics. *Annu. Rev. Physiol.* 81, 1-17. 10.1146/annurev-physiol-020518-114358 30256725

[JCS263830C26] Dorn, G. W. (2020). Mitofusins as mitochondrial anchors and tethers. *J. Mol. Cell. Cardiol.* 142, 146-153. 10.1016/j.yjmcc.2020.04.016 32304672 PMC7275906

[JCS263830C27] El Fissi, N., Rojo, M., Aouane, A., Karatas, E., Poliacikova, G., David, C., Royet, J. and Rival, T. (2018). Mitofusin gain and loss of function drive pathogenesis in Drosophila models of CMT2A neuropathy. *EMBO Rep.* 19, e45241. 10.15252/embr.201745241 29898954 PMC6073211

[JCS263830C28] Enkler, L. and Spang, A. (2024). Functional interplay of lipid droplets and mitochondria. *FEBS Lett.* 598, 1235-1251. 10.1002/1873-3468.14809 38268392

[JCS263830C29] Escobar-Henriques, M. and Joaquim, M. (2019). Mitofusins: disease gatekeepers and hubs in mitochondrial quality control by E3 ligases. *Front. Physiol.* 10, 517. 10.3389/fphys.2019.00517 31156446 PMC6533591

[JCS263830C30] Faitg, J., Davey, T., Turnbull, D. M., White, K. and Vincent, A. E. (2020). Mitochondrial morphology and function: two for the price of one! *J. Microsc.* 278, 89-106. 10.1111/jmi.12891 32277765

[JCS263830C31] Filadi, R., Pendin, D. and Pizzo, P. (2018). Mitofusin 2: from functions to disease. *Cell Death Dis.* 9, 1-13. 10.1038/s41419-017-0023-6 29491355 PMC5832425

[JCS263830C32] Franco, A., Kitsis, R. N., Fleischer, J. A., Gavathiotis, E., Kornfeld, O. S., Gong, G., Biris, N., Benz, A., Qvit, N., Donnelly, S. K. et al. (2016). Correcting mitochondrial fusion by manipulating mitofusin conformations. *Nature* 540, 74-79. 10.1038/nature20156 27775718 PMC5315023

[JCS263830C33] Freemont, P. S. (2000). Ubiquitination: RING for destruction? *Curr. Biol.* 10, R84-R87. 10.1016/S0960-9822(00)00287-6 10662664

[JCS263830C34] Friedman, J. R., Lackner, L. L., West, M., Dibenedetto, J. R., Nunnari, J. and Voeltz, G. K. (2011). ER tubules mark sites of mitochondrial division. *Science* 334, 358-362. 10.1126/science.1207385 21885730 PMC3366560

[JCS263830C35] Fu, M., St-Pierre, P., Shankar, J., Wang, P. T. C., Joshi, B. and Nabi, I. R. (2013). Regulation of mitophagy by the Gp78 E3 ubiquitin ligase. *Mol. Biol. Cell* 24, 1153-1162. 10.1091/mbc.e12-08-0607 23427266 PMC3623636

[JCS263830C36] Gan, X., Wang, J., Wang, C., Sommer, E., Kozasa, T., Srinivasula, S., Alessi, D., Offermanns, S., Simon, M. I. and Wu, D. (2012). PRR5L degradation promotes MTORC2-mediated PKCδ phosphorylation and cell migration downstream of Gα12. *Nat. Cell Biol.* 14, 686-696. 10.1038/ncb2507 22609986 PMC3389271

[JCS263830C37] Gan, X., Wang, C., Patel, M., Kreutz, B., Zhou, M., Kozasa, T. and Wu, D. (2013). Different Raf protein kinases mediate different signaling pathways to stimulate E3 ligase RFFL gene expression in cell migration regulation*. *J. Biol. Chem.* 288, 33978-33984. 10.1074/jbc.M113.477406 24114843 PMC3837137

[JCS263830C38] Gegg, M. E., Cooper, J. M., Chau, K.-Y., Rojo, M., Schapira, A. H. V. and Taanman, J.-W. (2010). Mitofusin 1 and mitofusin 2 are ubiquitinated in a PINK1/parkin-dependent manner upon induction of mitophagy. *Hum. Mol. Genet.* 19, 4861-4870. 10.1093/hmg/ddq419 20871098 PMC3583518

[JCS263830C39] Gomes, L. C., Benedetto, G. D. and Scorrano, L. (2011). During autophagy mitochondria elongate, are spared from degradation and sustain cell viability. *Nat. Cell Biol.* 13, 589-598. 10.1038/ncb2220 21478857 PMC3088644

[JCS263830C40] Gordaliza-Alaguero, I., Sànchez-Fernàndez-De-Landa, P., Radivojevikj, D., Villarreal, L., Arauz-Garofalo, G., Gay, M., Martinez-Vicente, M., Seco, J., Martín-Malpartida, P., Vilaseca, M. et al. (2025). Endogenous interactomes of MFN1 and MFN2 provide novel insights into interorganelle communication and autophagy. *Autophagy* 21, 957-978. 10.1080/15548627.2024.2440843 39675054 PMC12013434

[JCS263830C41] Guo, X., Chen, K.-H., Guo, Y., Liao, H., Tang, J. and Xiao, R.-P. (2007). Mitofusin 2 triggers vascular smooth muscle cell apoptosis via mitochondrial death pathway. *Circ. Res.* 101, 1113-1122. 10.1161/CIRCRESAHA.107.157644 17901359

[JCS263830C42] Hamdi, A., Roshan, T. M., Kahawita, T. M., Mason, A. B., Sheftel, A. D. and Ponka, P. (2016). Erythroid cell mitochondria receive endosomal iron by a “kiss-and-run” mechanism. *Biochim. Biophys. Acta* 1863, 2859-2867. 10.1016/j.bbamcr.2016.09.008 27627839

[JCS263830C43] Han, S., Zhao, F., Hsia, J., Ma, X., Liu, Y., Torres, S., Fujioka, H. and Zhu, X. (2021). The role of Mfn2 in the structure and function of endoplasmic reticulum-mitochondrial tethering in vivo. *J. Cell Sci.* 134, jcs253443. 10.1242/jcs.253443 34110411 PMC8277140

[JCS263830C44] Harper, C. S., White, A. J. and Lackner, L. L. (2020). The multifunctional nature of mitochondrial contact site proteins. *Curr. Opin. Cell Biol.* 65, 58-65. 10.1016/j.ceb.2020.02.010 32208350 PMC7771046

[JCS263830C45] Hu, L., Tang, D., Qi, B., Guo, D., Wang, Y., Geng, J., Zhang, X., Song, L., Chang, P., Chen, W. et al. (2024). Mfn2/Hsc70 complex mediates the formation of mitochondria-lipid droplets membrane contact and regulates myocardial lipid metabolism. *Adv. Sci.* 11, 2307749. 10.1002/advs.202307749 PMC11005711 38311582

[JCS263830C46] Huang, P., Yu, T. and Yoon, Y. (2007). Mitochondrial clustering induced by overexpression of the mitochondrial fusion protein Mfn2 causes mitochondrial dysfunction and cell death. *Eur. J. Cell Biol.* 86, 289-302. 10.1016/j.ejcb.2007.04.002 17532093

[JCS263830C47] Kim, H.-J., Nagano, Y., Choi, S. J., Park, S. Y., Kim, H., Yao, T.-P. and Lee, J.-Y. (2015). HDAC6 maintains mitochondrial connectivity under hypoxic stress by suppressing MARCH5/MITOL dependent MFN2 degradation. *Biochem. Biophys. Res. Commun.* 464, 1235-1240. 10.1016/j.bbrc.2015.07.111 26210454

[JCS263830C48] Kirisako, T., Kamei, K., Murata, S., Kato, M., Fukumoto, H., Kanie, M., Sano, S., Tokunaga, F., Tanaka, K. and Iwai, K. (2006). A ubiquitin ligase complex assembles linear polyubiquitin chains. *EMBO J.* 25, 4877-4887. 10.1038/sj.emboj.7601360 17006537 PMC1618115

[JCS263830C49] Kirshner, H., Aguet, F., Sage, D. and Unser, M. (2013). 3-D PSF fitting for fluorescence microscopy: implementation and localization application. *J. Microsc.* 249, 13-25. 10.1111/j.1365-2818.2012.03675.x 23126323

[JCS263830C50] Kitamura, T., Koshino, Y., Shibata, F., Oki, T., Nakajima, H., Nosaka, T. and Kumagai, H. (2003). Retrovirus-mediated gene transfer and expression cloning: powerful tools in functional genomics. *Exp. Hematol.* 31, 1007-1014. 10.1016/S0301-472X(03)00260-1 14585362

[JCS263830C51] Knott, A. B., Perkins, G., Schwarzenbacher, R. and Bossy-Wetzel, E. (2008). Mitochondrial fragmentation in neurodegeneration. *Nat. Rev. Neurosci.* 9, 505-518. 10.1038/nrn2417 18568013 PMC2711514

[JCS263830C52] Koshiba, T., Detmer, S. A., Kaiser, J. T., Chen, H., McCaffery, J. M. and Chan, D. C. (2004). Structural basis of mitochondrial tethering by mitofusin complexes. *Science* 305, 858-862. 10.1126/science.1099793 15297672

[JCS263830C53] Kumar, S., Acharya, T. K., Kumar, S., Mahapatra, P., Chang, Y.-T. and Goswami, C. (2024). TRPV4 modulation affects mitochondrial parameters in adipocytes and its inhibition upregulates lipid accumulation. *Life Sci.* 358, 123130. 10.1016/j.lfs.2024.123130 39413904

[JCS263830C54] Lam, J., Katti, P., Biete, M., Mungai, M., Ashshareef, S., Neikirk, K., Garza Lopez, E., Vue, Z., Christensen, T. A., Beasley, H. K. et al. (2021). A universal approach to analyzing transmission electron microscopy with ImageJ. *Cells* 10, 2177. 10.3390/cells10092177 34571826 PMC8465115

[JCS263830C55] Leboucher, G. P., Tsai, Y. C., Yang, M., Shaw, K. C., Zhou, M., Veenstra, T. D., Glickman, M. H. and Weissman, A. M. (2012). Stress-induced phosphorylation and proteasomal degradation of mitofusin 2 facilitates mitochondrial fragmentation and apoptosis. *Mol. Cell* 47, 547-557. 10.1016/j.molcel.2012.05.041 22748923 PMC3526191

[JCS263830C56] Li, W., Bengtson, M. H., Ulbrich, A., Matsuda, A., Reddy, V. A., Orth, A., Chanda, S. K., Batalov, S. and Joazeiro, C. A. P. (2008). Genome-wide and functional annotation of human E3 ubiquitin ligases identifies MULAN, a mitochondrial e3 that regulates the Organelle's dynamics and signaling. *PLoS ONE* 3, e1487. 10.1371/journal.pone.0001487 18213395 PMC2198940

[JCS263830C57] Liao, W., Xiao, Q., Tchikov, V., Fujita, K., Yang, W., Wincovitch, S., Garfield, S., Conze, D., El-Deiry, W. S., Schutze, S. et al. (2008). CARP-2 is an endosome-associated ubiquitin protein ligase for RIP and regulates TNF-induced NF-κB activation. *Curr. Biol.* 18, 641-649. 10.1016/j.cub.2008.04.017 18450452 PMC2587165

[JCS263830C58] Lim, K. L., Chew, K. C. M., Tan, J. M. M., Wang, C., Chung, K. K. K., Zhang, Y., Tanaka, Y., Smith, W., Engelender, S., Ross, C. A. et al. (2005). Parkin mediates nonclassical, proteasomal-independent ubiquitination of synphilin-1: implications for Lewy body formation. *J. Neurosci.* 25, 2002-2009. 10.1523/JNEUROSCI.4474-04.2005 15728840 PMC6726069

[JCS263830C59] Lin, C., Yan, J., Kapur, M. D., Norris, K. L., Hsieh, C., Huang, D., Vitale, N., Lim, K., Guan, Z., Wang, X. et al. (2022). Parkin coordinates mitochondrial lipid remodeling to execute mitophagy. *EMBO Rep.* 23, e55191. 10.15252/embr.202255191 36256516 PMC9724658

[JCS263830C60] Lin, Z., Akin, H., Rao, R., Hie, B., Zhu, Z., Lu, W., Smetanin, N., Verkuil, R., Kabeli, O., Shmueli, Y. et al. (2023). Evolutionary-scale prediction of atomic-level protein structure with a language model. *Science* 379, 1123-1130. 10.1126/science.ade2574 36927031

[JCS263830C61] Lupo, U., Sgarbossa, D. and Bitbol, A.-F. (2024). Pairing interacting protein sequences using masked language modeling. *Proc. Natl. Acad. Sci. USA* 121, e2311887121. 10.1073/pnas.2311887121 38913900 PMC11228504

[JCS263830C62] Maddi, E. R. and Natesh, R. (2021). Optimization strategies for expression and purification of soluble N-terminal domain of human centriolar protein SAS-6 in *Escherichia coli*. *Protein Expr. Purif.* 183, 105856. 10.1016/j.pep.2021.105856 33640460

[JCS263830C63] Mahdaviani, K., Benador, I. Y., Su, S., Gharakhanian, R. A., Stiles, L., Trudeau, K. M., Cardamone, M., Enríquez–Zarralanga, V., Ritou, E., Aprahamian, T. et al. (2017). Mfn2 deletion in brown adipose tissue protects from insulin resistance and impairs thermogenesis. *EMBO Rep.* 18, 1123-1138. 10.15252/embr.201643827 28539390 PMC5887905

[JCS263830C64] Mallick, K., Paul, S., Banerjee, S. and Banerjee, S. (2024). Lipid droplets and neurodegeneration. *Neuroscience* 549, 13-23. 10.1016/j.neuroscience.2024.04.014 38718916

[JCS263830C65] Mattie, S., Riemer, J., Wideman, J. G. and McBride, H. M. (2017). A new mitofusin topology places the redox-regulated C terminus in the mitochondrial intermembrane space. *J. Cell Biol.* 217, 507-515. 10.1083/jcb.201611194 29212658 PMC5800796

[JCS263830C66] McBride, H. M., Neuspiel, M. and Wasiak, S. (2006). Mitochondria: more than just a powerhouse. *Curr. Biol.* 16, R551-R560. 10.1016/j.cub.2006.06.054 16860735

[JCS263830C67] McDonald, E. R. and El-Deiry, W. S. (2004). Suppression of caspase-8- and -10-associated RING proteins results in sensitization to death ligands and inhibition of tumor cell growth. *Proc. Natl. Acad. Sci. USA* 101, 6170-6175. 10.1073/pnas.0307459101 15069192 PMC395941

[JCS263830C68] McLelland, G.-L., Goiran, T., Yi, W., Dorval, G., Chen, C. X., Lauinger, N. D., Krahn, A. I., Valimehr, S., Rakovic, A., Rouiller, I. et al. (2018). Mfn2 ubiquitination by PINK1/parkin gates the p97-dependent release of ER from mitochondria to drive mitophagy. *eLife* 7, e32866. 10.7554/eLife.32866 29676259 PMC5927771

[JCS263830C69] Meggouh, F., Bienfait, H. M. E., Weterman, M. A. J., De Visser, M. and Baas, F. (2006). Charcot-Marie-Tooth disease due to a de novo mutation of the RAB7 gene. *Neurology* 67, 1476-1478. 10.1212/01.wnl.0000240068.21499.f5 17060578

[JCS263830C70] Michel, M. A., Swatek, K. N., Hospenthal, M. K. and Komander, D. (2017). Ubiquitin linkage-specific affimers reveal insights into K6-linked ubiquitin signaling. *Mol. Cell* 68, 233-246.e5. 10.1016/j.molcel.2017.08.020 28943312 PMC5640506

[JCS263830C71] Mukherjee, R. and Chakrabarti, O. (2016). Regulation of Mitofusin1 by Mahogunin ring finger-1 and the proteasome modulates mitochondrial fusion. *Biochim. Biophys. Acta Mol. Cell Res.* 1863, 3065-3083. 10.1016/j.bbamcr.2016.09.022 27713096

[JCS263830C72] Müntjes, K., Devan, S. K., Reichert, A. S. and Feldbrügge, M. (2021). Linking transport and translation of mRNAs with endosomes and mitochondria. *EMBO Rep.* 22, e52445. 10.15252/embr.202152445 34402186 PMC8490996

[JCS263830C73] Naón, D., Hernández-Alvarez, M. I., Shinjo, S., Wieczor, M., Ivanova, S., Martins De Brito, O., Quintana, A., Hidalgo, J., Palacín, M., Aparicio, P. et al. (2023). Splice variants of mitofusin 2 shape the endoplasmic reticulum and tether it to mitochondria. *Science* 380, eadh9351. 10.1126/science.adh9351 37347868

[JCS263830C74] Nara, A., Inoue, A., Aoyama, Y. and Yazawa, T. (2023). The ultrastructural function of MLN64 in the late endosome–mitochondria membrane contact sites in placental cells. *Exp. Cell Res.* 429, 113668. 10.1016/j.yexcr.2023.113668 37245582

[JCS263830C75] Narendra, D., Tanaka, A., Suen, D.-F. and Youle, R. J. (2008). Parkin is recruited selectively to impaired mitochondria and promotes their autophagy. *J. Cell Biol.* 183, 795-803. 10.1083/jcb.200809125 19029340 PMC2592826

[JCS263830C76] Ng, Y. S. and Turnbull, D. M. (2016). Mitochondrial disease: genetics and management. *J. Neurol.* 263, 179-191. 10.1007/s00415-015-7884-3 26315846 PMC4723631

[JCS263830C77] Nunnari, J. and Suomalainen, A. (2012). Mitochondria: in sickness and in health. *Cell* 148, 1145-1159. 10.1016/j.cell.2012.02.035 22424226 PMC5381524

[JCS263830C78] Okiyoneda, T., Veit, G., Sakai, R., Aki, M., Fujihara, T., Higashi, M., Susuki-Miyata, S., Miyata, M., Fukuda, N., Yoshida, A. et al. (2018). Chaperone-independent peripheral quality control of CFTR by RFFL E3 ligase. *Dev. Cell* 44, 694-708.e7. 10.1016/j.devcel.2018.02.001 29503157 PMC6447300

[JCS263830C79] Ouyang, Q., Chen, Q., Ke, S., Ding, L., Yang, X., Rong, P., Feng, W., Cao, Y., Wang, Q., Li, M. et al. (2023). Rab8a as a mitochondrial receptor for lipid droplets in skeletal muscle. *Dev. Cell* 58, 289-305.e6. 10.1016/j.devcel.2023.01.007 36800997

[JCS263830C80] Parra, V., Eisner, V., Chiong, M., Criollo, A., Moraga, F., Garcia, A., Härtel, S., Jaimovich, E., Zorzano, A., Hidalgo, C. et al. (2008). Changes in mitochondrial dynamics during ceramide-induced cardiomyocyte early apoptosis. *Cardiovasc. Res.* 77, 387-397. 10.1093/cvr/cvm029 18006463

[JCS263830C81] Parra, V., Verdejo, H. E., Iglewski, M., Del Campo, A., Troncoso, R., Jones, D., Zhu, Y., Kuzmicic, J., Pennanen, C., Lopez-Crisosto, C. et al. (2013). Insulin stimulates mitochondrial fusion and function in cardiomyocytes via the Akt-mTOR-NFκB-Opa-1 signaling pathway. *Diabetes* 63, 75-88. 10.2337/db13-0340 24009260 PMC3868041

[JCS263830C82] Peng, W., Chen, S., Ma, J., Wei, W., Lin, N., Xing, J., Guo, W., Li, H., Zhang, L., Chan, K. et al. (2025). Endosomal trafficking participates in lipid droplet catabolism to maintain lipid homeostasis. *Nat. Commun.* 16, 1917. 10.1038/s41467-025-57038-8 39994216 PMC11850777

[JCS263830C83] Ponomareva, O. Y., Eliceiri, K. W. and Halloran, M. C. (2016). Charcot-Marie-tooth 2b associated Rab7 mutations cause axon growth and guidance defects during vertebrate sensory neuron development. *Neural Dev.* 11, 2. 10.1186/s13064-016-0058-x 26791407 PMC4721196

[JCS263830C84] Poole, A. C., Thomas, R. E., Yu, S., Vincow, E. S. and Pallanck, L. (2010). The mitochondrial fusion-promoting factor mitofusin is a substrate of the PINK1/Parkin pathway. *PLoS ONE* 5, e10054. 10.1371/journal.pone.0010054 20383334 PMC2850930

[JCS263830C85] Quintana-Cabrera, R. and Scorrano, L. (2023). Determinants and outcomes of mitochondrial dynamics. *Mol. Cell* 83, 857-876. 10.1016/j.molcel.2023.02.012 36889315

[JCS263830C86] Rambold, A. S., Kostelecky, B., Elia, N. and Lippincott-Schwartz, J. (2011). Tubular network formation protects mitochondria from autophagosomal degradation during nutrient starvation. *Proc. Natl. Acad. Sci. USA* 108, 10190-10195. 10.1073/pnas.1107402108 21646527 PMC3121813

[JCS263830C87] Ravindran, R., Velikkakath, A. K. G., Narendradev, N. D., Chandrasekharan, A., Santhoshkumar, T. R. and Srinivasula, S. M. (2022). Endosomal-associated RFFL facilitates mitochondrial clearance by enhancing PRKN/parkin recruitment to mitochondria. *Autophagy* 18, 2851-2864. 10.1080/15548627.2022.2052460 35373701 PMC9673925

[JCS263830C88] Ren, J., Wen, L., Gao, X., Jin, C., Xue, Y. and Yao, X. (2009). DOG 1.0: illustrator of protein domain structures. *Cell Res.* 19, 271-273. 10.1038/cr.2009.6 19153597

[JCS263830C89] Rocha, A. G., Franco, A., Krezel, A. M., Rumsey, J. M., Alberti, J. M., Knight, W. C., Biris, N., Zacharioudakis, E., Janetka, J. W., Baloh, R. H. et al. (2018). MFN2 agonists reverse mitochondrial defects in preclinical models of Charcot-Marie-Tooth disease type 2A. *Science* 360, 336-341. 10.1126/science.aao1785 29674596 PMC6109362

[JCS263830C90] Roder, K., Kabakov, A., Moshal, K. S., Murphy, K. R., Xie, A., Dudley, S., Turan, N. N., Lu, Y., MacRae, C. A. and Koren, G. (2019). Trafficking of the human ether-a-go-go-related gene (hERG) potassium channel is regulated by the ubiquitin ligase rififylin (RFFL). *J. Biol. Chem.* 294, 351-360. 10.1074/jbc.RA118.003852 30401747 PMC6322893

[JCS263830C91] Sakai, R., Fukuda, R., Unida, S., Aki, M., Ono, Y., Endo, A., Kusumi, S., Koga, D., Fukushima, T., Komada, M. et al. (2019). The integral function of the endocytic recycling compartment is regulated by RFFL-mediated ubiquitylation of Rab11 effectors. *J. Cell Sci.* 132, jcs228007. 10.1242/jcs.228007 30659120

[JCS263830C92] Sandoval, H., Yao, C.-K., Chen, K., Jaiswal, M., Donti, T., Lin, Y. Q., Bayat, V., Xiong, B., Zhang, K., David, G. et al. (2014). Mitochondrial fusion but not fission regulates larval growth and synaptic development through steroid hormone production. *eLife* 3, e03558. 10.7554/eLife.03558 25313867 PMC4215535

[JCS263830C93] Satoh, A. O., Fujioka, Y., Kashiwagi, S., Yoshida, A., Fujioka, M., Sasajima, H., Nanbo, A., Amano, M. and Ohba, Y. (2023). Interaction between PI3K and the VDAC2 channel tethers Ras-PI3K-positive endosomes to mitochondria and promotes endosome maturation. *Cell Rep.* 42, 112229. 10.1016/j.celrep.2023.112229 36906852

[JCS263830C94] Schägger, H. (2006). Tricine–SDS-PAGE. *Nat. Protoc.* 1, 16-22. 10.1038/nprot.2006.4 17406207

[JCS263830C95] Schindelin, J., Arganda-Carreras, I., Frise, E., Kaynig, V., Longair, M., Pietzsch, T., Preibisch, S., Rueden, C., Saalfeld, S., Schmid, B. et al. (2012). Fiji: an open-source platform for biological-image analysis. *Nat. Methods* 9, 676-682. 10.1038/nmeth.2019 22743772 PMC3855844

[JCS263830C96] Schneider, C. A., Rasband, W. S. and Eliceiri, K. W. (2012). NIH Image to ImageJ: 25 years of image analysis. *Nat. Methods* 9, 671-675. 10.1038/nmeth.2089 22930834 PMC5554542

[JCS263830C97] Sha, Z., Blyszcz, T., González-Prieto, R., Vertegaal, A. C. O. and Goldberg, A. L. (2019). Inhibiting ubiquitination causes an accumulation of SUMOylated newly synthesized nuclear proteins at PML bodies. *J. Biol. Chem.* 294, 15218-15234. 10.1074/jbc.RA119.009147 31285264 PMC6802522

[JCS263830C98] Shankar, J., Kojic, L. D., St-Pierre, P., Wang, P. T. C., Fu, M., Joshi, B. and Nabi, I. R. (2013). Raft endocytosis of AMF regulates mitochondrial dynamics through Rac1 signaling and the Gp78 ubiquitin ligase. *J. Cell Sci.* 126, 3295-3304. 10.1242/jcs.120162 23690547

[JCS263830C99] Sharma, R., Mondal, P. and Srinivasula, S. M. (2023). CARPs regulate STUB1 and its pathogenic mutants aggregation kinetics by mono-ubiquitination. *FEBS J.* 290, 3580-3594. 10.1111/febs.16766 36853170

[JCS263830C100] Sharma, R., Das, K. D. and Srinivasula, S. M. (2024). EGF-mediated Golgi dynamics and cell migration require CARP2. *Cell Rep.* 43, 114896. 10.1016/j.celrep.2024.114896 39441718

[JCS263830C101] Shcherbakova, D. M. and Verkhusha, V. V. (2013). Near-infrared fluorescent proteins for multicolor in vivo imaging. *Nat. Methods* 10, 751-754. 10.1038/nmeth.2521 23770755 PMC3737237

[JCS263830C102] Sheftel, A. D., Zhang, A.-S., Brown, C., Shirihai, O. S. and Ponka, P. (2007). Direct interorganellar transfer of iron from endosome to mitochondrion. *Blood* 110, 125-132. 10.1182/blood-2007-01-068148 17376890

[JCS263830C103] Shen, T., Zheng, M., Cao, C., Chen, C., Tang, J., Zhang, W., Cheng, H., Chen, K.-H. and Xiao, R.-P. (2007). Mitofusin-2 is a major determinant of oxidative stress-mediated heart muscle cell apoptosis *. *J. Biol. Chem.* 282, 23354-23361. 10.1074/jbc.M702657200 17562700

[JCS263830C104] Sidarala, V., Zhu, J., Levi-D'ancona, E., Pearson, G. L., Reck, E. C., Walker, E. M., Kaufman, B. A. and Soleimanpour, S. A. (2022). Mitofusin 1 and 2 regulation of mitochondrial DNA content is a critical determinant of glucose homeostasis. *Nat. Commun.* 13, 2340. 10.1038/s41467-022-29945-7 35487893 PMC9055072

[JCS263830C105] Stuppia, G., Rizzo, F., Riboldi, G., Del Bo, R., Nizzardo, M., Simone, C., Comi, G. P., Bresolin, N. and Corti, S. (2015). MFN2-related neuropathies: clinical features, molecular pathogenesis and therapeutic perspectives. *J. Neurol. Sci.* 356, 7-18. 10.1016/j.jns.2015.05.033 26143526

[JCS263830C106] Su, D., Ding, C., Wang, R., Qiu, J., Liu, Y., Tao, J., Luo, W., Weng, G., Yang, G. and Zhang, T. (2024). E3 ubiquitin ligase RBCK1 confers ferroptosis resistance in pancreatic cancer by facilitating MFN2 degradation. *Free Radic. Biol. Med.* 221, 136-154. 10.1016/j.freeradbiomed.2024.05.031 38763208

[JCS263830C107] Sugiura, A., Nagashima, S., Tokuyama, T., Amo, T., Matsuki, Y., Ishido, S., Kudo, Y., McBride, H. M., Fukuda, T., Matsushita, N. et al. (2013). MITOL regulates endoplasmic reticulum-mitochondria contacts via Mitofusin2. *Mol. Cell* 51, 20-34. 10.1016/j.molcel.2013.04.023 23727017

[JCS263830C108] Tanaka, A., Cleland, M. M., Xu, S., Narendra, D. P., Suen, D.-F., Karbowski, M. and Youle, R. J. (2010). Proteasome and p97 mediate mitophagy and degradation of mitofusins induced by Parkin. *J. Cell Biol.* 191, 1367-1380. 10.1083/jcb.201007013 21173115 PMC3010068

[JCS263830C109] Tang, F.-L., Liu, W., Hu, J.-X., Erion, J. R., Ye, J., Mei, L. and Xiong, W.-C. (2015). VPS35 deficiency or mutation causes dopaminergic neuronal loss by impairing mitochondrial fusion and function. *Cell Rep.* 12, 1631-1643. 10.1016/j.celrep.2015.08.001 26321632 PMC4565770

[JCS263830C110] Tang, W., Thundyil, J., Lim, G. G. Y., Tng, T. J. W., Yeow, S. Q. Z., Nair, A., Chai, C., Yao, T.-P. and Lim, K.-L. (2023). Parkin regulates neuronal lipid homeostasis through SREBP2-lipoprotein lipase pathway—implications for Parkinson's disease. *Hum. Mol. Genet.* 32, 1466-1482. 10.1093/hmg/ddac297 36519761 PMC10117165

[JCS263830C111] Tibbetts, M. D., Shiozaki, E. N., Gu, L., McDonald, E. R., El-Deiry, W. S. and Shi, Y. (2004). Crystal structure of a FYVE-type zinc finger domain from the caspase regulator CARP2. *Structure* 12, 2257-2263. 10.1016/j.str.2004.10.007 15576038

[JCS263830C112] Todkar, K., Chikhi, L. and Germain, M. (2019). Mitochondrial interaction with the endosomal compartment in endocytosis and mitochondrial transfer. *Mitochondrion* 49, 284-288. 10.1016/j.mito.2019.05.003 31100469

[JCS263830C113] Triolo, M., Wade, S., Baker, N. and Khacho, M. (2023). Evaluating mitochondrial length, volume, and cristae ultrastructure in rare mouse adult stem cell populations. *STAR Protoc.* 4, 102107. 10.1016/j.xpro.2023.102107 36853728 PMC9943866

[JCS263830C114] Tsubuki, S., Saito, Y., Tomioka, M., Ito, H. and Kawashima, S. (1996). Differential inhibition of calpain and proteasome activities by peptidyl aldehydes of di-leucine and tri-leucine. *J. Biochem. (Tokyo)* 119, 572-576. 10.1093/oxfordjournals.jbchem.a021280 8830056

[JCS263830C115] Verhoeven, K., Claeys, K. G., Züchner, S., Schröder, J. M., Weis, J., Ceuterick, C., Jordanova, A., Nelis, E., De Vriendt, E. and Van Hul, M. (2006). MFN2 mutation distribution and genotype/phenotype correlation in Charcot–Marie–Tooth type 2. *Brain* 129, 2093-2102. 10.1093/brain/awl126 16714318

[JCS263830C116] Wang, T. S., Coppens, I., Saorin, A., Brady, N. R. and Hamacher-Brady, A. (2020). Endolysosomal targeting of mitochondria is integral to BAX-mediated mitochondrial permeabilization during apoptosis signaling. *Dev. Cell* 53, 627-645.e7. 10.1016/j.devcel.2020.05.014 32504557 PMC7433306

[JCS263830C117] Yamano, K., Kikuchi, R., Kojima, W., Hayashida, R., Koyano, F., Kawawaki, J., Shoda, T., Demizu, Y., Naito, M., Tanaka, K. et al. (2020). Critical role of mitochondrial ubiquitination and the OPTN–ATG9A axis in mitophagy. *J. Cell Biol.* 219, e201912144. 10.1083/jcb.201912144 32556086 PMC7480101

[JCS263830C118] Yang, W., Rozan, L. M., McDdonald, E. R., Navaraj, A., Liu, J. J., Matthew, E. M., Wang, W., Dicker, D. T. and El-Deiry, W. S. (2007). CARPs are ubiquitin ligases that promote MDM2-independent p53 and phospho-p53ser20 degradation*. *J. Biol. Chem.* 282, 3273-3281. 10.1074/jbc.M610793200 17121812

[JCS263830C119] Youle, R. J. and Van Der Bliek, A. M. (2012). Mitochondrial fission, fusion, and stress. *Science* 337, 1062-1065. 10.1126/science.1219855 22936770 PMC4762028

[JCS263830C120] Yun, J., Puri, R., Yang, H., Lizzio, M. A., Wu, C., Sheng, Z.-H. and Guo, M. (2014). MUL1 acts in parallel to the PINK1/parkin pathway in regulating mitofusin and compensates for loss of PINK1/parkin. *eLife* 3, e01958. 10.7554/eLife.01958 24898855 PMC4044952

[JCS263830C121] Zadoorian, A., Du, X. and Yang, H. (2023). Lipid droplet biogenesis and functions in health and disease. *Nat. Rev. Endocrinol.* 19, 443-459. 10.1038/s41574-023-00845-0 37221402 PMC10204695

[JCS263830C122] Zhao, F., Wang, W., Wang, C., Siedlak, S. L., Fujioka, H., Tang, B. and Zhu, X. (2017). Mfn2 protects dopaminergic neurons exposed to paraquat both in vitro and in vivo: implications for idiopathic Parkinson's disease. *Biochim. Biophys. Acta Mol. Basis Dis.* 1863, 1359-1370. 10.1016/j.bbadis.2017.02.016 28215578 PMC5474135

[JCS263830C123] Ziviani, E., Tao, R. N. and Whitworth, A. J. (2010). Drosophila Parkin requires PINK1 for mitochondrial translocation and ubiquitinates Mitofusin. *Proc. Natl. Acad. Sci. USA* 107, 5018-5023. 10.1073/pnas.0913485107 20194754 PMC2841909

[JCS263830C124] Züchner, S., Mersiyanova, I. V., Muglia, M., Bissar-Tadmouri, N., Rochelle, J., Dadali, E. L., Zappia, M., Nelis, E., Patitucci, A., Senderek, J. et al. (2004). Mutations in the mitochondrial GTPase mitofusin 2 cause Charcot-Marie-Tooth neuropathy type 2A. *Nat. Genet.* 36, 449-451. 10.1038/ng1341 15064763

