# Supplementary material for: Endosomal RFFL ubiquitin ligase regulates mitochondrial morphology by targeting mitofusin 2
Source: J Cell Sci. 2025 Jun 20;138(12):jcs263830. doi: 10.1242/jcs.263830 (PMC12211564; doi:10.1242/jcs.263830)
Supplement: Supplementary information [file joces-138-263830-s1.pdf]

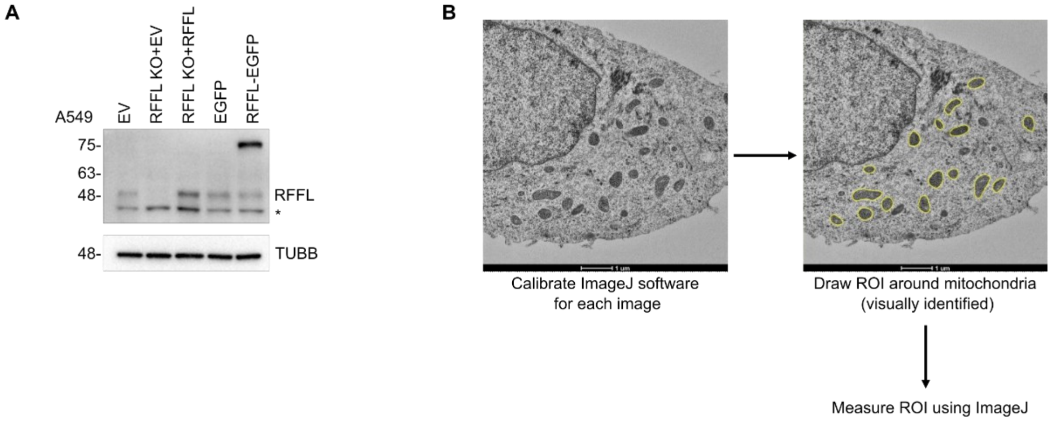

Fig. S1. (**A**) Western blot showing expression levels of RFFL in the cells used in this study. * represent the non-specific band. (**B**) Analysis workflow for EM image quantification.

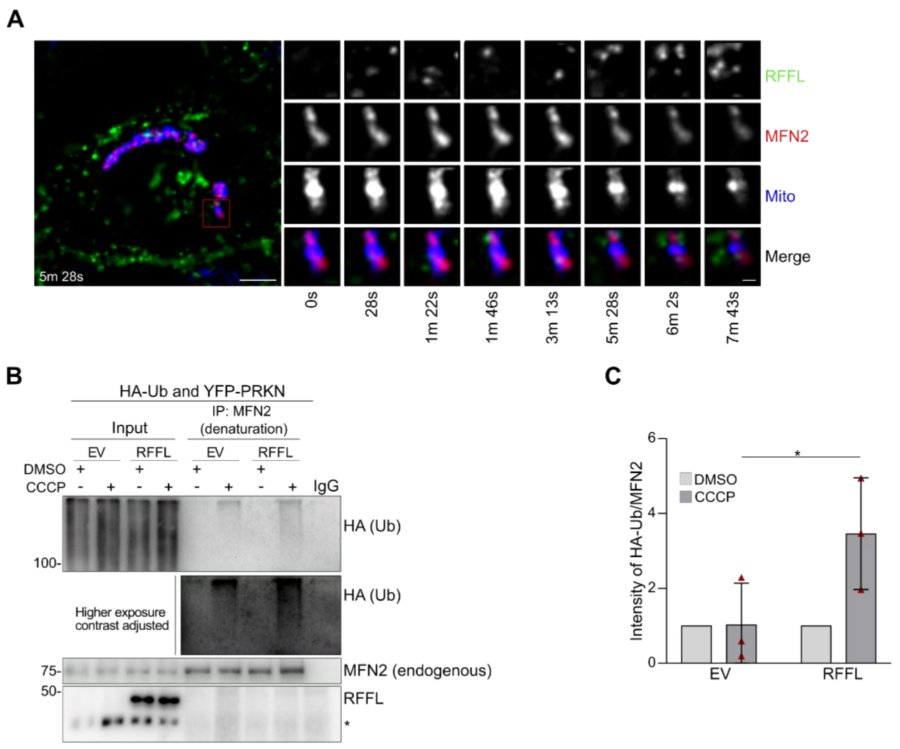

Fig. S2. (**A**) A549 cells stably expressing RFFL-EGFP were transfected with MFN2-iRFP670, and mitochondria were stained with MitoTracker Red CMXRos. Live time-lapse imaging was performed, and snapshots are shown here. Scale: 5 µm. Inset scale: 1 µm. (**B**) 293T cells stably expressing EV or WT RFFL were transiently transfected with HA-Ub and YFP-PRKN and treated with DMSO or 10µM CCCP for 45 minutes. Endogenous MFN2 was immunoprecipitated from these cell lysates using MFN2 specific antibodies under denaturation conditions. The samples were then immunoblotted with the indicated antibodies. * corresponds to a non-specific band recognized by anti-RFFL antibodies. (**C**) The bar graph shows the ratio of the intensity of the HA signal to the MFN2 signal in IP samples of (B). The error bar represents mean ± s.d. from three independent experiments. Statistical significance was calculated using a one-tailed unpaired t-test. p = 0.0430.

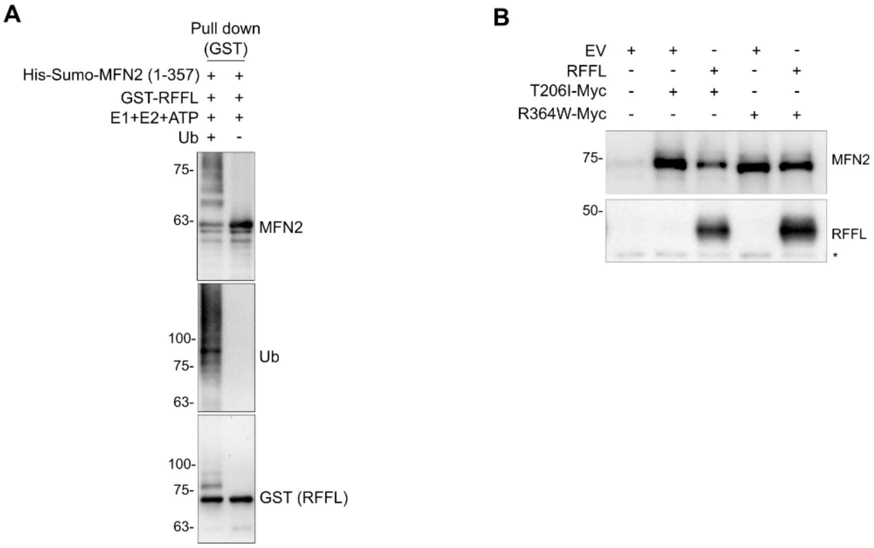

Fig. S3. (**A**) In vitro ubiquitination reaction using His-Sumo-MFN2 (1-357) bound to GST-RFFL with and without Ub. The modification only appears in the presence of Ub in the reaction. **B**) Western blot showing expression of transfected constructs in cells, used for Fig. 5L. *represents a non-specific band recognized by anti-RFFL antibodies.

Journal of Cell Science • Supplementary information

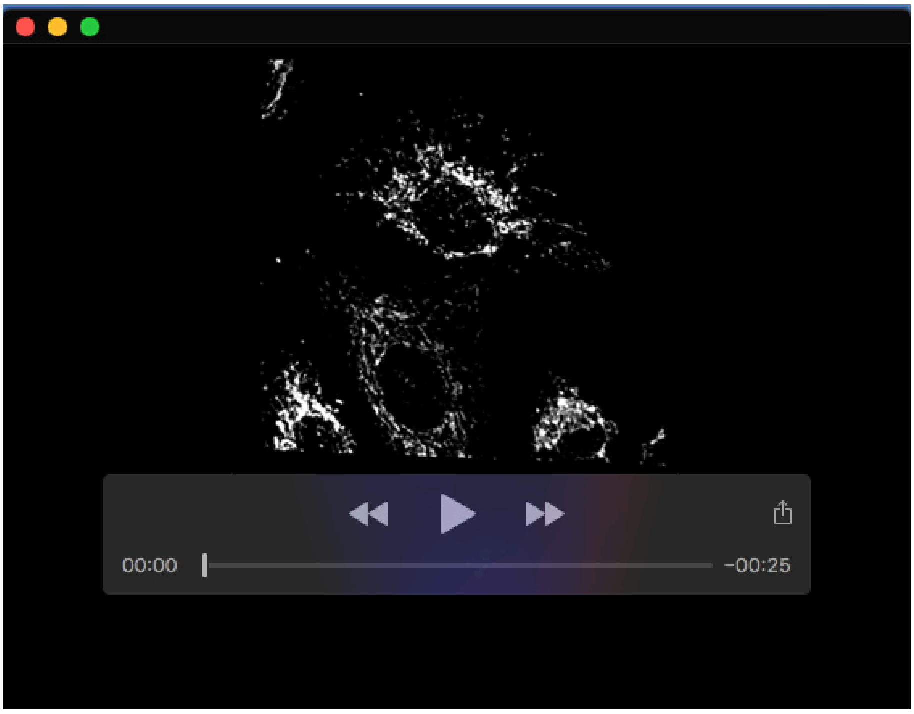

Movie 1. Three-dimensional reconstruction of mitochondria in A549 cells expressing EV. Cells were immunostained with an anti-TOMM20 antibody, and imaging was done with confocal microscopy. 3-D reconstruction of the images (Volume) was done from deconvoluted Z stacks. The contrast was adjusted for better visibility. Related to Fig. 1A.

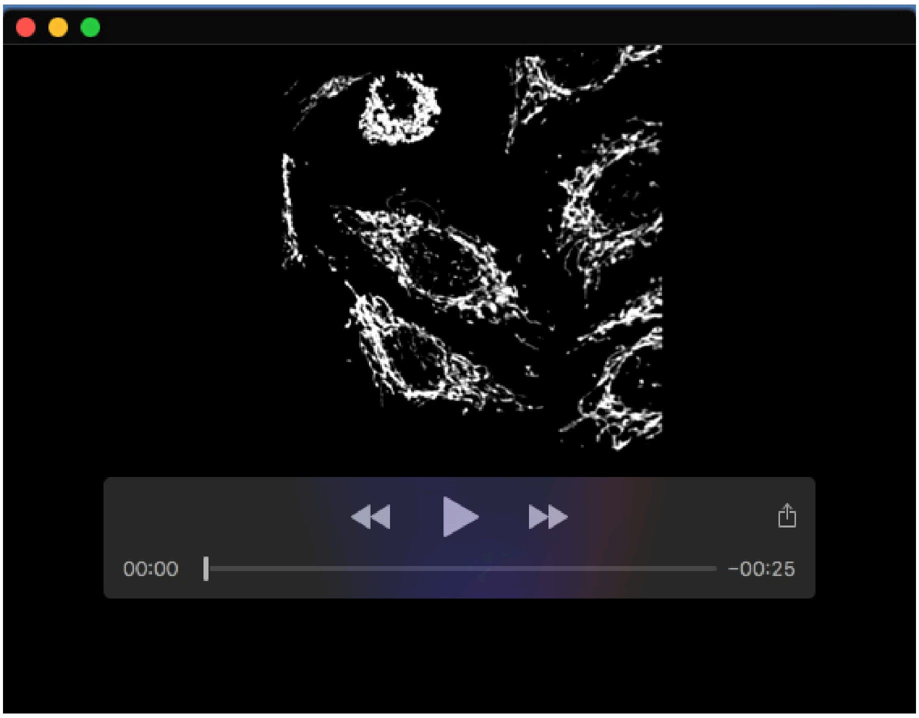

Movie 2. Three-dimensional reconstruction of mitochondria in A549 RFFL KO cells stably expressing EV. Cells were immunostained with an anti-TOMM20 antibody, and imaging was done with confocal microscopy. 3-D reconstruction (Volume) of the images was done from deconvoluted Z stacks. The contrast was adjusted for better visibility. Related to Fig. 1A.

Journal of Cell Science • Supplementary information

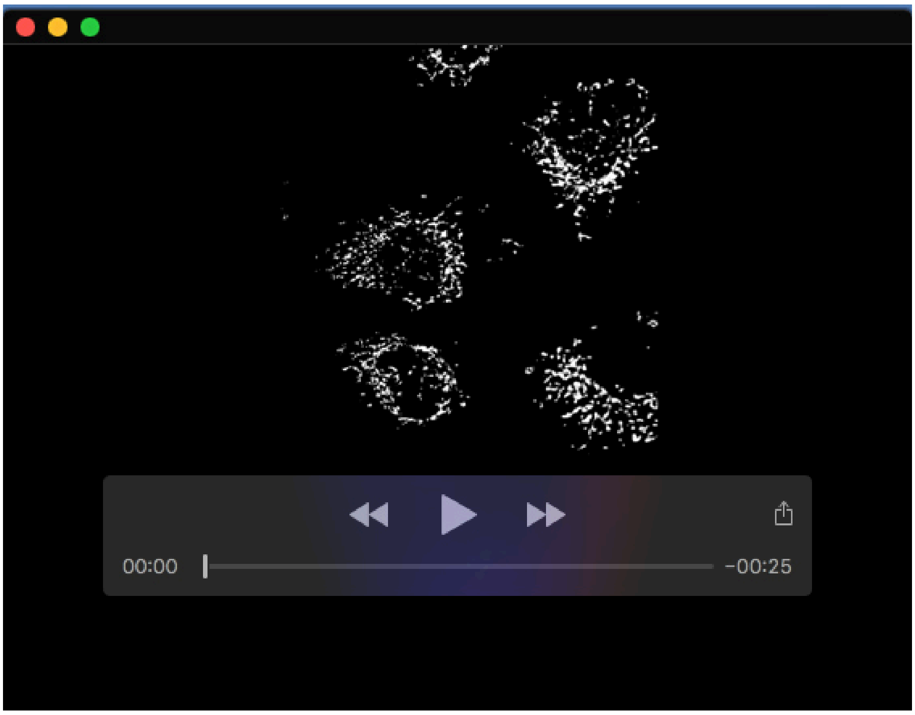

Movie 3. Three-dimensional reconstruction of mitochondria in A549 RFFL KO cells reconstituted with untagged WT RFFL. Cells were immunostained with an anti-TOMM20 antibody, and imaging was done with confocal microscopy. 3-D reconstruction (Volume) of the images was done from deconvoluted Z stacks. The contrast was adjusted for better visibility. Related to Fig. 1A.

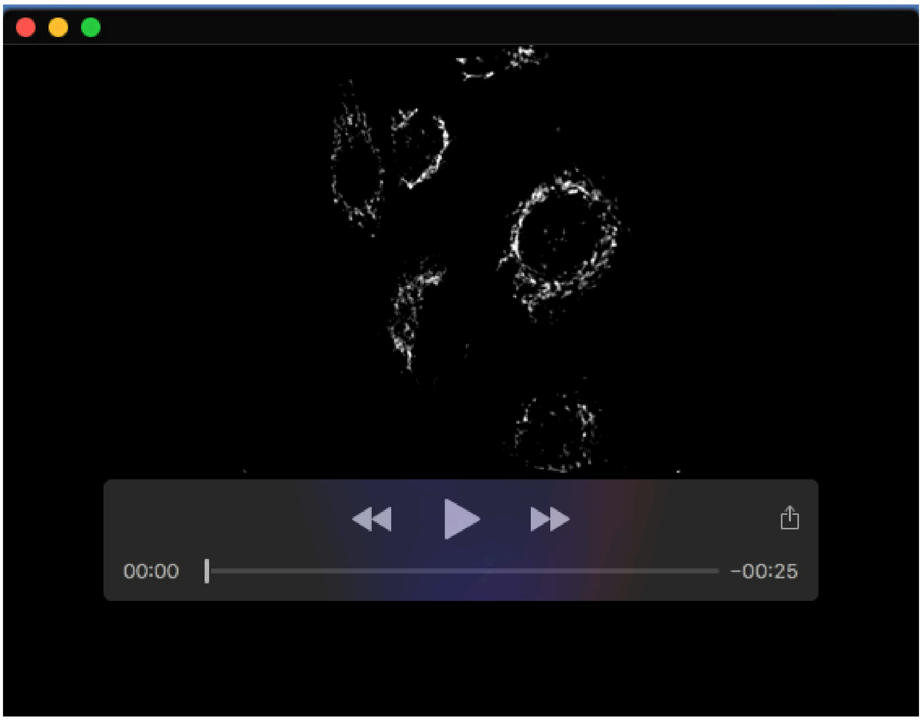

Movie 4. Three-dimensional reconstruction of mitochondria in A549 RFFL-EGFP stable cells. Cells were immunostained with an anti-TOMM20 antibody, and imaging was done with confocal microscopy. 3-D reconstruction (Volume) of the images was done from deconvoluted Z stacks. The contrast was adjusted for better visibility. Related to Fig. 1A.

Journal of Cell Science • Supplementary information

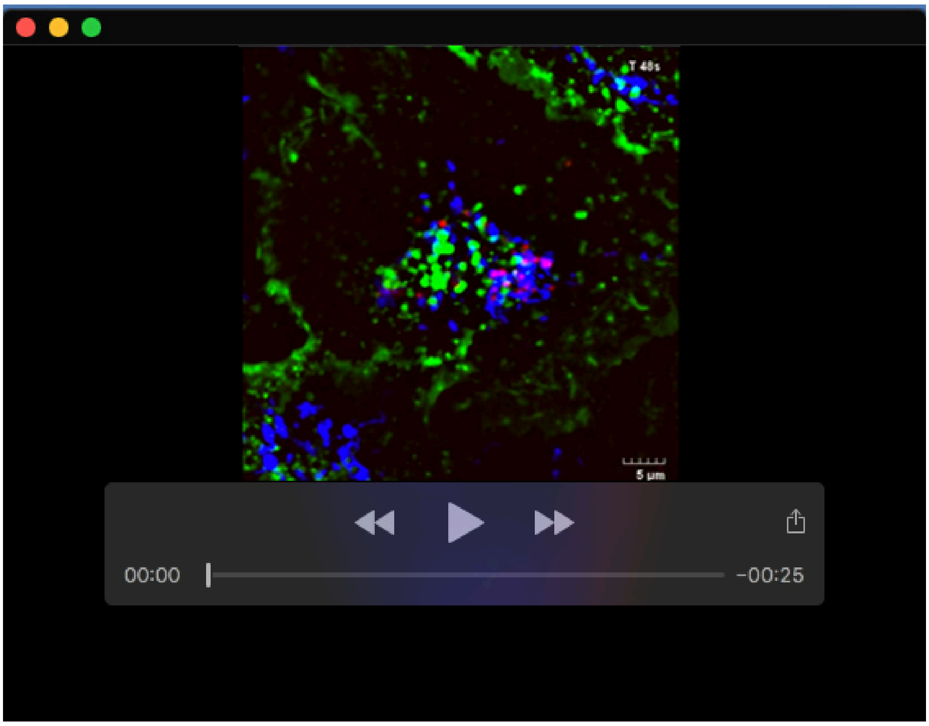

Movie 5. Dynamic association of RFFL vesicles with mitochondria and MFN2. Confocal time-lapse imaging of A549 cells stably expressing RFFL-EGFP (Green), transfected with MFN2-iRFP670 (Red) and stained for mitochondria with MitoTracker Red CMXRos (Blue) (10 frames per second). Related to Fig. 2D.

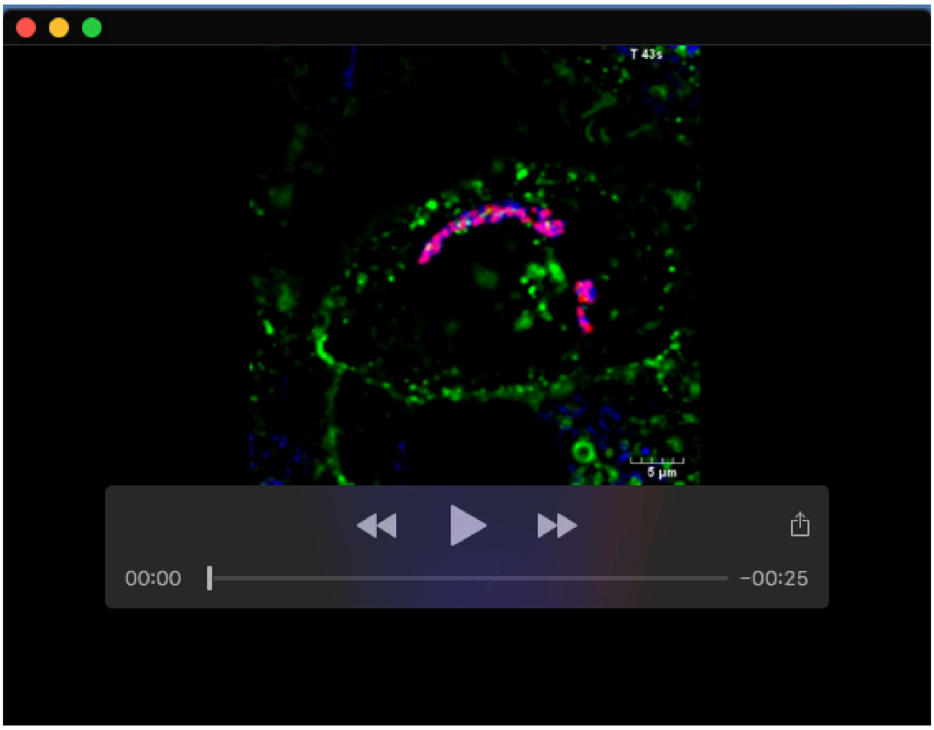

Movie 6. Dynamic association of RFFL vesicles with mitochondria and MFN2. Confocal time-lapse imaging of A549 cells stably expressing RFFL-EGFP (Green), transfected with MFN2-iRFP670 (Red) and stained for mitochondria with MitoTracker Red CMXRos (Blue) (10 frames per second). Related to Fig. S2A.
