# Supplementary material for: Endosomal RFFL ubiquitin ligase regulates mitochondrial morphology by targeting mitofusin 2
Source: J Cell Sci. 2025 Jun 20;138(12):jcs263830. doi: 10.1242/jcs.263830 (PMC12211564; doi:10.1242/jcs.263830)
Supplement: Supplementary file 1 [file joces-138-263830_review_history.pdf]

Journal of Cell Science

Endosomal RFFL ubiquitin ligase regulates mitochondrial morphology by targeting mitofusin 2
Nikhil Dev Narendradev, Rishith Ravindran, Parul Jain, Shikha Chaudhary, Anoop Kumar G. Velikkakath, Abyasree Sudharman, Adithya Janardhanan, Tapas Chandra Nag, Subhash Chandra Yadav and Srinivasa Murty Srinivasula
DOI: 10.1242/jcs.263830

Editor: Ana Garcia Saez

Review timeline

Original submission:	9 January 2025
Editorial decision:	11 February 2025
First revision received:	14 April 2025
Accepted:	18 May 2025

Original submission

First decision letter

MS ID#: jcs.263830

MS TITLE: Endosomal RFFL ubiquitin ligase regulates mitochondrial morphology by targeting Mitofusin 2

AUTHORS: Nikhil Dev Narendradev; Rishith Ravindran; Parul Jain; Shikha Chaudhary; Anoop Kumar G. Velikkakath; Abyasree Sudharman; Adithya Janardhanan; Tapas Chandra Nag; Subhash Chandra Yadav; Srinivasa Murty Srinivasula
ARTICLE TYPE: Research Article

Dear Dr Srinivasula,

We have now reached a decision on the above manuscript.

To see the reviewers' reports and a copy of this decision letter, please go to:

As you will see, the reviewers raise a number of substantial criticisms that prevent me from accepting the paper at this stage. They suggest, however, that a revised version might prove acceptable, if you can address their concerns. If you think that you can deal satisfactorily with the criticisms on revision, I would be pleased to see a revised manuscript. We would then return it to the reviewers.

Reviewer 1

SUMMARY OF THE ADVANCE MADE IN THIS PAPER AND ITS POTENTIAL SIGNIFICANCE TO THE FIELD
1. Shown a new level of regulation of MFN2
2. Importance of endosome-mitochondria contacts in regulation of MFN2
3. A new player in regulation of mitochondrial dynamics
Elegant and well executed study, makes an effort to understand how pathological mitochondrial hyperfusion may be rectified by modulating the fission-fusion dynamics.

SUGGESTIONS TO AUTHORS
1. Uniformity in statistical analysis is suggested.
2. A few Western blots need to be improved.

© 2025. Published by The Company of Biologists under the terms of the Creative Commons Attribution License (https://creativecommons.org/licenses/by/4.0/).

In the manuscript entitled “Endosomal RFFL ubiquitin ligase regulates mitochondrial morphology by targeting”, the authors have identified another ubiquitin E3 liagse which could regulate MFN2 levels. The ligase assigned this function is RFFL, an already established endosomal ligase. Through this work, the authors have not only shown another levels of regulation of MFN2, they have also touched upon the importance of endosomal-mitochondria contact sites in this regulation. Though this has not been explored in depth. Overall, this is an elegant study and if improved on the following points, should emerge as an important regulatory mechanism of mitochondrial dynamics.

Major points:

1) Fig1A: Data in Fig1C is complete with all the required controls. Fig1A should also include all these combinations.
2) Fig1B: Kruskal-Wallis test as indicated in the figure legend is not appropriate for comparison between EV and RFFL KO+RFFL group. Hence a more appropriate statistical analysis is required. Data representation with percentage of cells exhibiting hyperfused mitochondria would be useful in this context.
3) Fig1C: In a single experimental setup two different RFFL constructs have been used and compared. Justify the logic.
4) In Fig1 authors have used different statistical analyses for very similar experiments (non parametric Kruskal-Wallis test in Fig1B but in Fig1D and Fig1E they went for parametric one way ANOVA) – what is the scientific logic for this? If not, then please maintain one appropriate analysis.
5) Fig1D & 1E: RFFL KO+ RFFL group showed more fragmentation than WT. Is it an artifact of overexpression, rather than a rescue? Please show by Western blots, the levels of overexpression in all figures.
6) Fig2C: One way ANOVA is not appropriate test particularly for comparing EV and RFFL KO+RFFL group.
7) Fig2E: As authors indicated that RFFL could interact with MFN2 even in constitutive condition; a similar experiment without PRKN overexpression is also required.
8) Why are there no visible protein bands of RFFL in EV of Input section; the present blot has bands only in RFFL OE condition?
9) Fig2G: Authors have used extracts from YPF-PRKN stably expressing cells but, GFP (YFP-PRKN) seems to have differential expression. This blot is saturated and needs to be improved.
10) Fig2H: Authors analysed the data using twoway ANOVA as they indicated in the figure legend. Although their experimental design and comparing pairs of interest are well enough for applying one way ANOVA.
11) Fig5A: Blots need to improved. No quantitation has been presented.
12) Fig5B: No RFFL endogenous band is visible in EV section. Positive control for MG132 is required.
13) Fig5H: It seems RFFL OE in T206I cells completely rescue WT phenotype. Authors have used ‘mean area per mito’ here for exhibiting mitochondrial structure unlike ‘mean volume per mito’ in Fig1B. Scientific logic for doing so?

Minor point:

Discussion section is lengthy with many redundant statements. This section could be trimmed and improved.

<u>Reviewer 2</u>

SUMMARY OF THE ADVANCE MADE IN THIS PAPER AND ITS POTENTIAL SIGNIFICANCE TO THE FIELD

This is a very strong and interesting manuscript presented for peer review by investigating a novel finding that RFFL directly interacts with MFN2. The authors use two standard microscopic techniques including quantification and interpretation. I find this article to be both timely and novel. This study provides new insights into mitochondrial dynamics and protein interactions, which

© 2025. Published by The Company of Biologists under the terms of the Creative Commons Attribution License (https://creativecommons.org/licenses/by/4.0/).

will help advance the field by broadening our understanding of post translational modifications in mitochondria biology. My suggestions are below.

SUGGESTIONS TO AUTHORS

1. Overall, my primary concern with Figure 1 is the selection of quantifications used to represent enlarged mitochondria. As demonstrated by these authors and others in the field, mitochondria are dynamic organelles that constantly undergo morphological changes. To properly assess mitochondrial structure, particularly the presence of enlarged mitochondria, additional analyses should be included.
2. Fig. 1:The authors currently present only volume, area per mitochondrion, and perimeter per mitochondrion. However, mitochondrial length and width should also be quantified to provide a more comprehensive analysis. Since the manuscript discusses multi-organelle contact sites, mitochondrial interactions with endosomes could also be quantified to further support the findings.Sampling mitochondria near endosomes would be very interesting and could further drive home the points addressed in figure 1. A few publications to consider for reference: PMID: 34571826 , PMID: 32277765, PMID: 24009260)
3. Fig1:Additionally, the authors could utilize TEM imaging at higher magnification to observe and analyze cristae (PMID: 36853728, PMID: 28096338). Cristae morphology through scoring, can be quantified as a functional readout of mitochondrial integrity and function. Based on the images in Figure 1C, the EM appears to be of sufficient quality to visualize cristae, suggesting that quantitative analysis of cristae morphology should be feasible—particularly for the enlarged mitochondria. These data could provide insights into the physiological relevance of this phenotype.
4. Fig.1:Previous studies have shown that perturbations in cristae morphology result in enlarged and dysfunctional mitochondria (PMID: 26857402; PMID: 31284597). Given the evidence presented in the manuscript, the enlarged mitochondria observed may be linked to metabolic function. This could be further supported by performing Seahorse experiments in conjunction with cristae scoring, which would provide functional metabolic data to complement the EM observations.
5. Although the manuscript focuses solely on MFN2, it would be valuable to include a discussion of MFN1, as it is the next candidate (MLM) in Figure 2A and a homologous GTPase. Both MFN1 and MFN2 localize to the outer mitochondrial membrane (OMM) and regulate fusion. MFN1 has been shown to exhibit higher GTPase activity than MFN2, suggesting that it may be more effective in promoting mitochondrial fusion and contributing to the enlarged mitochondria phenotype. Western blotting for MFN1 could provide insight into whether its endogenous expression is affected by RFFL and whether MFN1 serves as a compensatory protein in facilitating RFFL-mediated ubiquitination of MFN2.
6. In line 140, please explain the readouts you will use for mitochondrial morphology. 2D isn't sufficient. The supplemental 3D figures may need to be incorporated into the main figures to drive home this point.

Minor comments:

7. The manuscript discusses multiple multi-organelle contact sites in both the introduction and discussion, including mitochondria-endosome, mitochondria-ER, and mitochondria-lipid interactions. However, these interactions are not assessed in the figures. While the primary focus is on RFFL-endosome interactions with MFN2, it would be interesting to explore the direct effects on lipid dynamics, given that mitochondrial morphology is highly influenced by lipid composition.
8. Endosomal trafficking is heavily dependent on calcium signaling. It would be beneficial for the authors to address how calcium signaling—both from the cytosol and mitochondrial calcium stores—affects endosomal signaling and RFFL expression in regulating MFN2. Additionally, it would be valuable to determine whether RFFL is sensitive to Ca^{2+}-induced conformational changes that might influence its ubiquitination function.
9. Since endosomes physically interact with mitochondria to facilitate lipid exchange, further investigation into how the described processes affect mitochondrial-lipid interactions could provide deeper insight into the mechanisms contributing to the enlarged mitochondria phenotype.

First revision

<u>Author response to reviewers' comments</u>

© 2025. Published by The Company of Biologists under the terms of the Creative Commons Attribution License (https://creativecommons.org/licenses/by/4.0/).

Reviewer 1

SUMMARY OF THE ADVANCE MADE IN THIS PAPER AND ITS POTENTIAL SIGNIFICANCE TO THE FIELD

1. Shown a new level of regulation of MFN2
2. Importance of endosome-mitochondria contacts in regulation of MFN2
3. A new player in regulation of mitochondrial dynamics

Elegant and well executed study, makes an effort to understand how pathological mitochondrial hyperfusion may be rectified by modulating the fission-fusion dynamics.

SUGGESTIONS TO AUTHORS

1. Uniformity in statistical analysis is suggested.
2. A few Western blots need to be improved.

In the manuscript entitled “Endosomal RFFL ubiquitin ligase regulates mitochondrial morphology by targeting”, the authors have identified another ubiquitin E3 liagse which could regulate MFN2 levels. The ligase assigned this function is RFFL, an already established endosomal ligase. Through this work, the authors have not only shown another levels of regulation of MFN2, they have also touched upon the importance of endosomal-mitochondria contact sites in this regulation. Though this has not been explored in depth. Overall, this is an elegant study and if improved on the following points, should emerge as an important regulatory mechanism of mitochondrial dynamics.

Response: We thank the reviewer for the constructive feedback. In the revised manuscript, the concerns were addressed with new experimental evidence wherever required. Point-by-point responses follow.

Major points:

1) Fig1A: Data in Fig1C is complete with all the required controls. Fig1A should also include all these combinations.

Response: We thank the reviewer for pointing out this oversight. We have now performed additional experiments as suggested. The revised manuscript includes the data with all the controls, with new images and quantification (Fig. 1A-F).

2) Fig1B: Kruskal-Wallis test as indicated in the figure legend is not appropriate for comparison between EV and RFFL KO+RFFL group. Hence a more appropriate statistical analysis is required. Data representation with percentage of cells exhibiting hyperfused mitochondria would be useful in this context.

Response: We thank the reviewer for the suggestion. We have now used unpaired two-tailed Student's t-test with Bonferroni's correction for the analysis and maintained uniformity across the paper. As suggested, the percentage of cells exhibiting normal, fragmented, and hyperfused/interconnected mitochondria was calculated, and the data was included in Figure 1F. As suggested by the second reviewer, quantification data showing the length and width of mitochondria are also included in Figures 1D and 1E. Publications with methodologies employed for analyses and representation were also cited.

3) Fig1C: In a single experimental setup two different RFFL constructs have been used and compared. Justify the logic.

Response: We appreciate the reviewer’s comment. We have used two types of cells with RFFL over-expression in this experiment: (1). A549 wild-type cells stably expressing RFFL-EGFP under CMV promoter, and (2). A549 knockout cells reconstituted with RFFL cDNA under a weak promoter. Though both types express RFFL, they differ in the extent of RFFL protein expression and also one of them is expressed in tandem with EGFP, while the other is expressed without any tag. Hence, we

© 2025. Published by The Company of Biologists under the terms of the Creative Commons Attribution License (https://creativecommons.org/licenses/by/4.0/).

used both cell types for our experiments to assess the effects of the tag and the protein expression levels, if any. Strengthening our hypothesis, data from both cell types clearly supports a role for RFFL in mitochondrial morphology.

4) In Fig1 authors have used different statistical analyses for very similar experiments (non parametric Kruskal-Wallis test in Fig1B but in Fig1D and Fig1E they went for parametric one way ANOVA) – what is the scientific logic for this? If not, then please maintain one appropriate analysis.

Response: We appreciate the reviewer's comment. We have now applied unpaired two-tailed Student's t-test with Bonferroni's correction for the analysis and maintained uniformity across the paper. Legends were updated appropriately.

5) Fig1D & 1E: RFFL KO+ RFFL group showed more fragmentation than WT. Is it an artifact of overexpression, rather than a rescue? Please show by Western blots, the levels of overexpression in all figures.

Response: We appreciate the reviewer's critical observation. As suggested, immunoblots showing the RFFL protein in stable cells were included in Fig. S1A. As discussed in response to query #3, to avoid any artifacts, the KO was reconstituted with the RFFL without any tag under a weak promoter. The observed differences between WT, RFFL KO, RFFL KO+ RFFL, and RFFL-EGFP could as well be attributable to protein expression level. Nonetheless, we believe the differences further establish RFFL as a regulator of mitochondrial dynamics.

6) Fig2C: One way ANOVA is not an appropriate test particularly for comparing EV and RFFL KO+RFFL group.

Response: We thank the reviewer for the suggestion. We have now performed a two-tailed unpaired t-test with Bonferroni's correction. The figures and legends are changed accordingly.

7) Fig2E: As authors indicated that RFFL could interact with MFN2 even in constitutive condition; a similar experiment without PRKN overexpression is also required.

Response: As suggested, we have now repeated denaturation IP experiments from lysates of cells that don't express PRKN and found RFFL-mediated MFN2 ubiquitination even without PRKN expression. The data from the new experiment is included in Fig. 2E and 2F, whereas the previous figures with PRKN expression are now presented in Fig. S2B and S2C.

8) Why are there no visible protein bands of RFFL in EV of Input section; the present blot has bands only in RFFL OE condition?

Response: The expression of endogenous RFFL protein is more detectable in A549 than in HEK293T cells. The observation of the reviewer is correct, as we also noted differences in the band intensity. It is because of the extent of exposure and the number of stripping-reprobing the membrane has been subjected to. The exogenous RFFL level is always higher than the endogenous protein.

9) Fig2G: Authors have used extracts from YPF-PRKN stably expressing cells but, GFP (YFP-PRKN) seems to have differential expression. This blot is saturated and needs to be improved.

Response: We appreciate the reviewer's critical feedback. These YFP-PRKN stable cells are generated by retroviral integration of YFP-PRKN to A549 WT and RFFL KO cells separately. Hence, slight differences in expression levels is not unexpected. To detect modified PRKN bands in response to CCCP treatment, higher exposure was used in the original figure. As per the suggestion, we have also included a new panel with lower exposure for GFP (YFP-PRKN), which showed minimal

© 2025. Published by The Company of Biologists under the terms of the Creative Commons Attribution License (https://creativecommons.org/licenses/by/4.0/).

difference in PRKN expression, if any (Fig. 2G).

10) Fig2H: Authors analysed the data using two-way ANOVA as they indicated in the figure legend. Although their experimental design and comparing pairs of interest are well enough for applying one way ANOVA.

Response: We thank the reviewer for the comment. As suggested, to bring uniformity across the paper, we felt it is more appropriate to use unpaired two-tailed Student's t-test with Bonferroni's correction and performed the same for the analysis. Even with different statistical tests/analyses, the conclusion remained unaltered.

11) Fig5A: Blots need to be improved. No quantitation has been presented.

Response: We have now replaced the figure with better blots. Quantification data along with statistical analysis (unpaired two-tailed Student's t-test with Bonferroni's correction) is also included (Fig. 5A, B).

12) Fig5B: No RFFL endogenous band is visible in the EV section. Positive control for MG132 is required.

Response: We thank the reviewer for pointing this out. As mentioned above in response to point 8, depending on the extent of exposure and the number of stripping-reprobing done on the membrane, the intensity of the endogenous protein band varied. As suggested, as a positive control for MG132, the samples were evaluated by probing with anti-Ub antibody. As expected, MG132 treatment resulted in the accumulation of more poly-Ub smears. (McMahon et al., 2024; Schimmel et al., 2008; Sulkshane et al., 2020). A new panel of Ub probing is now included in the figure (Fig. 5H).

13) Fig5H: It seems RFFL OE in T206I cells completely rescue WT phenotype. Authors have used 'mean area per mito' here for exhibiting mitochondrial structure unlike 'mean volume per mito' in Fig1B. Scientific logic for doing so?

Response: We sincerely appreciate the reviewer's observation. For this analysis, we used live cells and acquired images from a single focal plane. Z-stack imaging was avoided to minimize potential erroneous results in 3D volume measurements that could result from mitochondrial movement. Based on evidence presented in Fig. 1, in this context not much difference in the pattern mean area vs volume is expected.

Minor point:

Discussion section is lengthy with many redundant statements. This section could be trimmed and improved.

Response: We thank the reviewer for the suggestion. As recommended, the Discussion section is revised.

Reviewer 2

SUMMARY OF THE ADVANCE MADE IN THIS PAPER AND ITS POTENTIAL SIGNIFICANCE TO THE FIELD

This is a very strong and interesting manuscript presented for peer review by investigating a novel finding that RFFL directly interacts with MFN2. The authors use two standard microscopic techniques including quantification and interpretation. I find this article to be both timely and novel. This study provides new insights into mitochondrial dynamics and protein interactions, which

© 2025. Published by The Company of Biologists under the terms of the Creative Commons Attribution License (https://creativecommons.org/licenses/by/4.0/).

will help advance the field by broadening our understanding of post translational modifications in mitochondria biology. My suggestions are below.

Response: We thank the reviewer for the constructive feedback. In the revised manuscript, the concerns were addressed with new experimental evidence wherever required. Point-by-point responses follow:

SUGGESTIONS TO AUTHORS

1. Overall, my primary concern with Figure 1 is the selection of quantifications used to represent enlarged mitochondria. As demonstrated by these authors and others in the field, mitochondria are dynamic organelles that constantly undergo morphological changes. To properly assess mitochondrial structure, particularly the presence of enlarged mitochondria, additional analyses should be included.

Response: We thank the reviewer for the comment. As suggested, we have now performed additional quantification analyses by measuring the length, width, and % of cells with enlarged mitochondria and Feret's diameter (in the case of EM images), approaches that are employed by different groups in the field (Chaudhry et al., 2020; Das et al., 2022a; Das et al., 2022b; Triolo et al., 2023). All these different measurements consistently revealed changes in mitochondrial morphology (Fig. 1B-F, 1H-J) in RFFL-dependent manner, thus allying the concerns.

2. Fig. 1:The authors currently present only volume, area per mitochondrion, and perimeter per mitochondrion. However, mitochondrial length and width should also be quantified to provide a more comprehensive analysis. Since the manuscript discusses multi-organelle contact sites, mitochondrial interactions with endosomes could also be quantified to further support the findings. Sampling mitochondria near endosomes would be very interesting and could further drive home the points addressed in figure 1. A few publications to consider for reference: PMID: 34571826 , PMID: 32277765, PMID: 24009260).

Response: We appreciate the reviewer's comments. As mentioned above, we have now also included measurements of mitochondrial length and width (Fig. 1D, 1E).

With regard to sampling of mitochondria near endosomes, our group using confocal microscopy imaging earlier demonstrated a substantial association between RFFL endosomes and mitochondria in CCCP-treated cells, which also showed limited constitutive colocalization in control (DMSO treated) cells (Fig. 1 in Ravindran et al, 2022). In the same study, we also observed that RFFL localized mitochondria are also positive for RAB5B and RAB7A (Fig. 2 in Ravindran et al, 2022). However, deletion of RFFL didn't significantly affect RAB7A association with mitochondria (Fig. S2B in Ravindran et al, 2022), but delayed PRKN recruitment to the mitochondria.

Furthermore, in this study, we observed that a variant of RFFL (C5,6A) that is unable to associate with endosomes also failed to degrade MFN2 (Fig. 5C). Based on these data and findings presented in this study, we believe that the association of RFFL with mitochondria is required for MFN2 degradation, but RFFL itself is dispensable for endosomal association (e.g., RAB7A) with mitochondria.

3. Fig1: Additionally, the authors could utilize TEM imaging at higher magnification to observe and analyze cristae (PMID: 36853728, PMID: 28096338). Cristae morphology through scoring, can be quantified as a functional readout of mitochondrial integrity and function. Based on the images in Figure 1C, the EM appears to be of sufficient quality to visualize cristae, suggesting that quantitative analysis of cristae morphology should be feasible—particularly for the enlarged mitochondria. These data could provide insights into the physiological relevance of this phenotype.

Response: We agree with the reviewer that the cristae readout can give further information. In fact, we have acquired TEM images for cristae. As the number of images with sufficient quality of cristae is not up to the level of our satisfaction, we feel we need to standardize the sample preparation to get high-quality images and to have a meaningful conclusion. The images are given

© 2025. Published by The Company of Biologists under the terms of the Creative Commons Attribution License (https://creativecommons.org/licenses/by/4.0/).

below for the consideration of the reviewer, though they have not been included or mentioned in the manuscript.

NOTE: an image provided in confidence for the reviewers has been removed

However, as suggested by the reviewer (point # 7&9), we also investigated mitochondrial-lipid interactions. The data (Fig. 6), clearly demonstrated the accumulation of neutral lipids in RFFL-EGFP expressing cells. In these cells, though the number and the area of lipid droplets (LDs) are more, the colocalization between LDs and mitochondria was observed to be less. This observation is consistent with earlier reports of increased lipid content with reduced MFN2 protein levels (Cai et al., 2024; Hu et al., 2024). While these results are interesting, more experiments are needed to understand the physiological relevance of these findings.

4. Fig.1:Previous studies have shown that perturbations in cristae morphology result in enlarged and dysfunctional mitochondria (PMID: 26857402; PMID: 31284597). Given the evidence presented in the manuscript, the enlarged mitochondria observed may be linked to metabolic function. This could be further supported by performing Seahorse experiments in conjunction with cristae scoring, which would provide functional metabolic data to complement the EM observations.

Response: We thank the reviewer for the suggestion, but unfortunately, we were unable to complete the metabolic functional analysis. However, as the reviewer predicted, we found changes in lipid accumulation and LDs vs mitochondrial association in RFFL-expressing cells, revealing hitherto unexpected crosstalk between RFFL-mitochondria and lipid homeostasis.

5. Although the manuscript focuses solely on MFN2, it would be valuable to include a discussion of MFN1, as it is the next candidate (MLM) in Figure 2A and a homologous GTPase. Both MFN1 and MFN2 localize to the outer mitochondrial membrane (OMM) and regulate fusion. MFN1 has been shown to exhibit higher GTPase activity than MFN2, suggesting that it may be more effective in promoting mitochondrial fusion and contributing to the enlarged mitochondria phenotype. Western blotting for MFN1 could provide insight into whether its endogenous expression is affected by RFFL and whether MFN1 serves as a compensatory protein in facilitating RFFL-mediated ubiquitination of MFN2.

Response: We thank the reviewer for the suggestion. We have now evaluated the effect of RFFL expression on MFN1 as well as DRP1. As the reviewer pointed out, RFFL expression also reduced the MFN1 protein level. This effect of RFFL is specific to MFNs, as under the same experimental conditions, we could not see any effect on mitochondrial fusion protein DRP1. This new data is now included in Fig. 5D-G. Because of the non-availability of MFN1 antibody immediately, we could not check the RFFL effect on endogenous MFN1 protein levels.

6. In line 140, please explain the readouts you will use for mitochondrial morphology. 2D isn't sufficient. The supplemental 3D figures may need to be incorporated into the main figures to drive home this point.

Response: We apologize for the lack of clarity. In the revised manuscript, additional parameters, the length and the width, in addition to the volume of mitochondria, were also measured and quantification of the results are included in Fig. 1. References for methodologies employed for analyses and representation were also cited. We have now replaced images of Fig. 1 with images based on 3D rendering and the same is also shown in supplementary movies (Movies 1-4).

Minor comments:

7. The manuscript discusses multiple multi-organelle contact sites in both the introduction and discussion, including mitochondria-endosome, mitochondria-ER, and mitochondria-lipid interactions. However, these interactions are not assessed in the figures. While the primary focus is on RFFL-endosome interactions with MFN2, it would be interesting to explore the direct effects on

© 2025. Published by The Company of Biologists under the terms of the Creative Commons Attribution License (https://creativecommons.org/licenses/by/4.0/).

lipid dynamics, given that mitochondrial morphology is highly influenced by lipid composition.

Response: We thank the reviewer for the insightful comments. As suggested, we now have assessed the lipid dynamics in A549 cells expressing RFFL-EGFP or EGFP alone using HCS LipidTOX Deep Red, a dye that stains neutral lipids. Confocal images of these cells showed significant differences in lipid staining between the cells with or without RFFL expression with more LDs in RFFL expressing cells (Fig. 6A, B). Moreover, the colocalization between LDs and mitochondria (stained with MitoTracker Red CMXRos) was found to be less in RFFL expressing cells. These observations are consistent with results reported earlier in cells with reduced MFN2 (Boutant et al., 2017; Cai et al., 2024; Mahdaviani et al., 2017). These results are now included as Fig. 6 in the revised manuscript.

8. Endosomal trafficking is heavily dependent on calcium signaling. It would be beneficial for the authors to address how calcium signaling—both from the cytosol and mitochondrial calcium stores—affects endosomal signaling and RFFL expression in regulating MFN2. Additionally, it would be valuable to determine whether RFFL is sensitive to Ca^{2+}-induced conformational changes that might influence its ubiquitination function.

Response: We appreciate the reviewer's insightful suggestions and appreciate the importance of addressing potential cross-talk between endosomal RFFL and calcium signaling. We are trying to comprehensively address this aspect along with sensitive to Ca^{2+}-induced conformational changes that might influence RFFL ubiquitination function and hope the reviewer will agree that results from such investigations further our understanding but are beyond the scope of this study.

9. Since endosomes physically interact with mitochondria to facilitate lipid exchange, further investigation into how the described processes affect mitochondrial-lipid interactions could provide deeper insight into the mechanisms contributing to the enlarged mitochondria phenotype.

Response: We thank the reviewer for the insightful comments. As mentioned in response to comment #7, in initial experiments, we observed a significant difference in the accumulation of neutral lipids in RFFL expressing cells than control cells and also a decrease in colocalization between LDs and mitochondria. We have included these interesting observations in Figure 6. More investigations are required to get a deeper insight into the mechanisms involved.

References

Boutant, M., Kulkarni, S. S., Joffraud, M., Ratajczak, J., Valera-Alberni, M., Combe, R., Zorzano, A. and Cantó, C. (2017). Mfn2 is critical for brown adipose tissue thermogenic function. *EMBO J.* **36**, 1543–1558.

Cai, Z., Luo, W., Wang, H., Zhu, R., Yuan, Y., Zhan, X., Xie, M., Zhuang, H., Chen, H., Xu, Y., et al. (2024). MFN2 suppresses the accumulation of lipid droplets and the progression of clear cell renal cell carcinoma. *Cancer Sci.* **115**, 1791–1807.

Chaudhry, A., Shi, R. and Luciani, D. S. (2020). A pipeline for multidimensional confocal analysis of mitochondrial morphology, function, and dynamics in pancreatic β-cells. *Am. J. Physiol.-Endocrinol. Metab.* **318**, E87–E101.

Das, R., Das, S., Chakrabarti, S. and Chakrabarti, O. (2022a). CMT2A-linked mitochondrial hyperfusion-driving mutant MFN2 perturbs ER-mitochondrial associations and Ca2+ homeostasis. *Biol. Cell* **114**, 309–319.

Das, R., Kamal, I. M., Das, S., Chakrabarti, S. and Chakrabarti, O. (2022b). MITOL-mediated DRP1 ubiquitylation and degradation promotes mitochondrial hyperfusion in a CMT2A-linked MFN2 mutant. *J. Cell Sci.* **135**, jcs257808.

© 2025. Published by The Company of Biologists under the terms of the Creative Commons Attribution License (https://creativecommons.org/licenses/by/4.0/).

Hu, L., Tang, D., Qi, B., Guo, D., Wang, Y., Geng, J., Zhang, X., Song, L., Chang, P., Chen, W., et al. (2024). Mfn2/Hsc70 Complex Mediates the Formation of Mitochondria-Lipid Droplets Membrane Contact and Regulates Myocardial Lipid Metabolism. *Adv. Sci.* **11**, 2307749.

Mahdaviani, K., Benador, I. Y., Su, S., Gharakhanian, R. A., Stiles, L., Trudeau, K. M., Cardamone, M., Enríquez-Zarralanga, V., Ritou, E., Aprahamian, T., et al. (2017). Mfn2 deletion in brown adipose tissue protects from insulin resistance and impairs thermogenesis. *EMBO Rep.* 18, 1123–1138.

McMahon, A., Zhao, J. and Yan, S. (2024). Ubiquitin-mediated regulation of APE2 protein abundance. *J. Biol. Chem.* **300**, 107337.

Ravindran, R., Velikkakath, A. K. G., Narendradev, N. D., Chandrasekharan, A., Santhoshkumar, T. R. and Srinivasula, S. M. (2022). Endosomal-associated RFFL facilitates mitochondrial clearance by enhancing PRKN/parkin recruitment to mitochondria. *Autophagy* **18**, 2851–2864.

Schimmel, J., Larsen, K. M., Matic, I., Hagen, M. van, Cox, J., Mann, M., Andersen, J. S. and Vertegaal, A. C. O. (2008). The Ubiquitin-Proteasome System Is a Key Component of the SUMO-2/3 Cycle. *Mol. Cell. Proteomics* **7**, 2107–2122.

Sulkshane, P., Duek, I., Ram, J., Thakur, A., Reis, N., Ziv, T. and Glickman, M. H. (2020). Inhibition of proteasome reveals basal mitochondrial ubiquitination. *J. Proteomics* **229**, 103949.

Triolo, M., Wade, S., Baker, N. and Khacho, M. (2023). Evaluating mitochondrial length, volume, and cristae ultrastructure in rare mouse adult stem cell populations. *STAR Protoc.* **4**, 102107.

Second decision letter

MS ID#: jcs.263830R1

MS Title: Endosomal RFFL ubiquitin ligase regulates mitochondrial morphology by targeting Mitofusin 2

Authors: Nikhil Dev Narendradev; Rishith Ravindran; Parul Jain; Shikha Chaudhary; Anoop Kumar G. Velikkakath; Abyasree Sudharman; Adithya Janardhanan; Tapas Chandra Nag; Subhash Chandra Yadav; Srinivasa Murty Srinivasula
Article Type: Research Article

Dear Dr Srinivasula,

I am happy to tell you that your manuscript has been accepted for publication in Journal of Cell Science, pending standard publication integrity checks. It was accepted on 18 May 2025.

Reviewer 1

SUMMARY OF THE ADVANCE MADE IN THIS PAPER AND ITS POTENTIAL SIGNIFICANCE TO THE FIELD

The authors have explored the regulation of MFN2 and its modulation of mitochondrial morphology. The authors have tried top understand the importance of endosome-mitochondria contact sites in the regulation of mitochondrial morphology.

SUGGESTIONS TO AUTHORS

© 2025. Published by The Company of Biologists under the terms of the Creative Commons Attribution License (https://creativecommons.org/licenses/by/4.0/).

All points addressed and the manuscript may be accepted.

© 2025. Published by The Company of Biologists under the terms of the Creative Commons Attribution License (https://creativecommons.org/licenses/by/4.0/).
